# Promoting Partner Testing and Couples Testing through Secondary Distribution of HIV Self-Tests: A Randomized Clinical Trial

**DOI:** 10.1371/journal.pmed.1002166

**Published:** 2016-11-08

**Authors:** Samuel H. Masters, Kawango Agot, Beatrice Obonyo, Sue Napierala Mavedzenge, Suzanne Maman, Harsha Thirumurthy

**Affiliations:** 1 Department of Health Policy and Management, Gillings School of Global Public Health, University of North Carolina at Chapel Hill, Chapel Hill, North Carolina, United States of America; 2 Impact Research and Development Organization, Kisumu, Kenya; 3 RTI International, San Francisco, California, United States of America; 4 Department of Health Behavior, Gillings School of Global Public Health, University of North Carolina at Chapel Hill, Chapel Hill, North Carolina, United States of America; 5 Carolina Population Center, University of North Carolina at Chapel Hill, Chapel Hill, North Carolina, United States of America; Massachusetts General Hospital, UNITED STATES

## Abstract

**Background:**

Achieving higher rates of partner HIV testing and couples testing among pregnant and postpartum women in sub-Saharan Africa is essential for the success of combination HIV prevention, including the prevention of mother-to-child transmission. We aimed to determine whether providing multiple HIV self-tests to pregnant and postpartum women for secondary distribution is more effective at promoting partner testing and couples testing than conventional strategies based on invitations to clinic-based testing.

**Methods and Findings:**

We conducted a randomized trial in Kisumu, Kenya, between June 11, 2015, and January 15, 2016. Six hundred antenatal and postpartum women aged 18–39 y were randomized to an HIV self-testing (HIVST) group or a comparison group. Participants in the HIVST group were given two oral-fluid-based HIV test kits, instructed on how to use them, and encouraged to distribute a test kit to their male partner or use both kits for testing as a couple. Participants in the comparison group were given an invitation card for clinic-based HIV testing and encouraged to distribute the card to their male partner, a routine practice in many health clinics. The primary outcome was partner testing within 3 mo of enrollment. Among 570 participants analyzed, partner HIV testing was more likely in the HIVST group (90.8%, 258/284) than the comparison group (51.7%, 148/286; difference = 39.1%, 95% CI 32.4% to 45.8%, *p <* 0.001). Couples testing was also more likely in the HIVST group than the comparison group (75.4% versus 33.2%, difference = 42.1%, 95% CI 34.7% to 49.6%, *p <* 0.001). No participants reported intimate partner violence due to HIV testing. This study was limited by self-reported outcomes, a common limitation in many studies involving HIVST due to the private manner in which self-tests are meant to be used.

**Conclusions:**

Provision of multiple HIV self-tests to women seeking antenatal and postpartum care was successful in promoting partner testing and couples testing. This approach warrants further consideration as countries develop HIVST policies and seek new ways to increase awareness of HIV status among men and promote couples testing.

**Trial Registration:**

ClinicalTrials.gov NCT02386215.

## Abstract 2

In a randomized clinical trial, Harsha Thirumurthy and colleagues compare the efficacy of providing HIV self-tests to pregnant and postpartum women to conventional strategies using invitations to clinic-based testing.

## Author Summary

**Why Was This Study Done?:**

- Despite progress in recent years, men in sub-Saharan Africa have lower HIV testing rates than women, and nearly half of all HIV-positive individuals remain unaware of their HIV status; this represents a key barrier to meeting the UNAIDS 90-90-90 targets for HIV elimination.
- Among pregnant and postpartum women, male partner testing has been encouraged for HIV prevention purposes but remains uncommon in many countries.
- Recent WHO guidelines on HIV testing services have acknowledged the potential of HIV self-testing as a means to increase testing uptake among those not reached by other testing services, and have called for additional research to explore the potential benefits of self-testing.
- “Secondary distribution” of self-tests by women visiting health facilities has the potential to increase access to testing among their male sexual partners and to promote results disclosure and safer sexual decision-making.

**What Did the Researchers Do and Find?:**

- We conducted a randomized trial among 600 women attending antenatal and postpartum clinics in western Kenya to determine whether providing them multiple HIV self-tests is more effective at promoting partner testing and couples testing than a conventional strategy based on partner invitations to clinic-based testing.
- In the three months of follow-up, male partner testing was reported to have occurred for 90% of women in the HIV self-testing group but only 52% of women in the comparison group, a difference that was statistically significant.
- Couples testing was more than twice as likely among women who received multiple HIV self-tests than women who received invitations for their partners (75% in the HIV self-testing group versus 33% in the comparison group).
- The self-testing intervention was safe and effective at promoting partner HIV testing among women whose partners had not tested in the past 12 months.

**What Do These Findings Mean?:**

- The promising results of this study suggest that secondary distribution of self-tests warrants further consideration as countries develop HIV self-testing policies and seek new ways to promote male partner testing and couples testing.
- Additional research is recommended to explore uptake of clinic-based confirmatory testing among partners who self-test HIV-positive, which was beyond the scope of this research.
- The feasibility of secondary distribution strategies among other populations and settings should be explored, along with the potential of HIV self-tests to facilitate safer sexual behaviors.

## Introduction

Low uptake of HIV testing services in sub-Saharan Africa (SSA) is among the key barriers to meeting the 90-90-90 targets established by UNAIDS and to improving the effectiveness of HIV treatment as prevention. HIV testing among men in particular remains low in many countries, as does knowledge of HIV status among HIV-infected persons [1]. Door-to-door testing and mobile testing strategies have moved testing services out of health facilities and into communities, overcoming barriers related to clinic-based testing and, subsequently, increasing testing coverage. However, despite these advancements, there remains a need for novel interventions that can promote testing among men and other hard-to-reach populations [2,3].

In addition to increasing HIV testing uptake among men, achieving higher rates of couples testing can also contribute to HIV prevention efforts. Low uptake of couples testing is particularly concerning in light of data indicating that four out of every ten new HIV infections occur within stable heterosexual partnerships and that the majority of persons in sero-discordant relationships are unaware of their HIV status [4]. The benefits of couples testing may include safer sexual behavior in couples [5], higher uptake of interventions such as antiretroviral therapy (ART) for HIV-positive partners [6], and pre-exposure prophylaxis (PrEP) among HIV-negative partners in sero-discordant relationships, as well as increased uptake of and adherence to prevention of mother-to-child transmission (PMTCT) interventions [7–9]. Given the need to achieve better PMTCT outcomes and prevent new infections in couples, a number of countries have sought to promote partner testing and couples testing among pregnant and postpartum women [10]. However, efforts to encourage pregnant and postpartum women to refer their male partners for HIV testing have had limited success [11,12]. The barriers to testing among male partners have included stigma, fear of prognosis, lack of awareness of HIV risk, inconvenience, fear of disclosure, transportation costs, opportunity costs such as time off from work, and behavioral factors such as a tendency to delay behaviors with immediate costs and delayed benefits [13,14].

HIV self-testing (HIVST) is a promising approach that addresses many barriers associated with clinic-based HIV testing and has had high acceptability in SSA [15–17]. Self-tests enable individuals to test themselves for HIV privately and at their own convenience. Simple oral-fluid-based tests have achieved high sensitivity and specificity, with some studies also having shown that the tests can be used accurately by individuals [18]. A number of countries in SSA have developed policies for implementation and support of HIVST [19,20], with Kenya being the first country in SSA to include HIVST in its national testing guidelines [21]. Recent research in Kenya has also demonstrated the acceptability and feasibility of a novel “secondary distribution” strategy that seeks to promote HIV testing among men and in couples through provision of multiple self-tests to women seeking health services [22].

We conducted a randomized trial in Kenya among women receiving antenatal care (ANC) or postpartum care (PPC) services to test whether the provision of multiple self-tests to women for distribution to their partners can increase uptake of male partner testing and couples testing.

## Methods

### Ethics Statement

The study received approval from the Scientific and Ethics Review Unit at the Kenya Medical Research Institute and the Office of Human Research Ethics at the University of North Carolina at Chapel Hill.

### Study Setting

The study was conducted in urban and peri-urban areas within Kisumu County, Kenya. Adult HIV prevalence in Kisumu County is 19.3% [23], the third highest among the counties in the country. Women visiting ANC and PPC clinics were recruited from three health facilities in Kisumu.

### Study Design and Participants

Trained research assistants screened and enrolled women seeking ANC or PPC at the three facilities, in a private location away from regular clinic activities. Women were given the opportunity to enroll in the study if they met the following eligibility criteria: were 18–39 y of age, reported that their primary partner was not known to be HIV-positive or had not tested in the past 6 mo, resided in or around Kisumu County, and had no intention of leaving the area within 3 mo. In addition, at the ANC clinic eligibility was limited to women with gestation age ≤ 20 wk, and at the PPC clinic eligibility was limited to women who had given birth in the past 6 wk to 12 mo. Following the provision of written informed consent, participants were administered a baseline questionnaire that measured demographic characteristics, sexual behavior, HIV testing history, and partner characteristics. All study staff received ethical training on research with human participants.

### Randomization Procedures

Participants were randomized in a 1:1 ratio using balanced block randomization (block size 20) to an HIVST group or a comparison group. Sealed randomization envelopes were offered to participants sequentially, and these revealed the study group assignment to the participant and study staff simultaneously.

### Intervention

Participants in the HIVST group received two oral-fluid-based rapid HIV tests (OraQuick Rapid HIV-1/2 Antibody Test, OraSure Technologies). Each test was accompanied with an instruction sheet that described step-by-step self-testing procedures in multiple languages. Study staff also provided the participants with a brief demonstration of how to use the test. Participants were encouraged to distribute a test kit to their male partner or to use both test kits to undertake couples testing if they felt comfortable doing so; they were also counseled on how to talk to their partners about HIV testing, the possibility of adverse reactions associated with suggesting HIV testing to their partner, learning their partner’s HIV status, and disclosing their own HIV status. Following Kenya’s 2015 HIV testing services guidelines [19], participants were informed about the need to seek clinic-based confirmatory testing if a positive (reactive) self-test result was obtained, and an invitation card for confirmatory testing at a clinic in the study area was included with each test.

Participants in the comparison group were counseled on the importance of partner testing and provided with an invitation card to give to their partner for HIV testing at a study clinic. The use of invitation cards to promote male partner testing is currently standard practice in many facilities. The cards mentioned the importance of testing, listed the health facility where the participant was enrolled, and encouraged the male partner to get tested at the study facility.

Both groups received information on where to seek advice and assistance for clinical, counseling, and legal support in case of intimate partner violence (IPV). They also were given a study phone number to call in case they had questions or needed advice about clinic-based testing or self-testing, or IPV or other adverse events.

### Follow-Up Assessments

Follow-up data collection occurred over a 3-mo period. Participants were contacted each month to determine if they had distributed a self-test kit to their sexual partner (HIVST group) or if their partner had sought HIV testing at a clinic (comparison group). Research assistants scheduled and conducted an in-person follow-up interview with participants who reported having distributed a test to their partner or who reported that their partner sought clinic-based testing, while participants who had not done so or were not reached at 1 and 2 mo were interviewed at 3 mo. If participants were unable to meet with research assistants, a follow-up phone interview was conducted. Participants in both groups were asked whether their partner had been tested for HIV since study enrollment.

### Statistical Analyses

The unit of analysis was the study participant. All outcomes were self-reported by study participants. The primary, prespecified outcome was whether the primary partner of the participant had an HIV test within 3 mo of enrollment, which was determined from the follow-up survey question: “Has your partner had an HIV test since you were enrolled in the study?” The primary analysis compared this outcome in the HIVST and comparison groups using an unadjusted modified Poisson regression with robust standard errors [24]. Our original analysis plan proposed estimation of a logistic regression model, but ultimately we selected a modified Poisson model because risk ratios can be easier to interpret than odds ratios. We chose to present both the absolute risk differences between the two study groups and the risk ratios from modified Poisson regressions. Participants who were not successfully followed up were not included in the analyses as it was not possible to determine the primary and secondary outcomes for them.

In secondary analyses we examined the impact of the intervention on the following six outcomes reported by participants: (1) discussion of HIV testing with partner, (2) couples testing, (3) couples testing among participants whose partner tested for HIV, (4) awareness of partner’s HIV test result, (5) awareness of partner’s HIV test result among participants whose partner tested for HIV, and (6) partner’s HIV test result. Discussion of HIV testing was defined as having occurred if the participant reported that she and her partner had talked about HIV testing since enrollment in the study. Couples testing was defined as having occurred when a participant reported that she had tested together with her partner at the same time. Awareness of partner’s HIV test result was defined as the participant having learned her partner’s HIV status. Additionally, we examined whether partners of participants in the HIVST group who tested positive sought confirmatory testing and whether partners in both groups who received a positive result were reported to be in care at the time of follow-up. We also assessed IPV at baseline and follow-up using questions adapted from the Kenya Demographic and Health Survey [25] that asked whether participants experienced physical, emotional, verbal, or sexual violence from their partner. Participants were coded as having experienced IPV if they responded affirmatively to any of the IPV questions. Survey questions used to measure study outcomes are reported in S1 Table.

In order to determine whether there were differences in intervention effectiveness in certain populations, we estimated modified Poisson regression models among participants who were enrolled at each of the three health facilities, among those whose primary partner had tested for HIV in the 12 mo prior to enrollment or not, and among those who had experienced IPV in the 12 mo prior to enrollment or not. All statistical tests were two-sided, and significance level was set at *p* < 0.05. No adjustment was made for multiple testing since the secondary analyses were considered exploratory. Statistical analyses were performed using Stata 14.1.

The planned sample size for the study was 600, with 300 participants in each study group. Power calculations assuming a two-sided unadjusted independent proportions test indicated that with a sample size of 300 per study group and 20% uptake of partner testing in the comparison group, there would be 80% power to detect a difference in partner testing as small as 10%.

## Results

### Participant Recruitment and Flow

**Fig 1:**
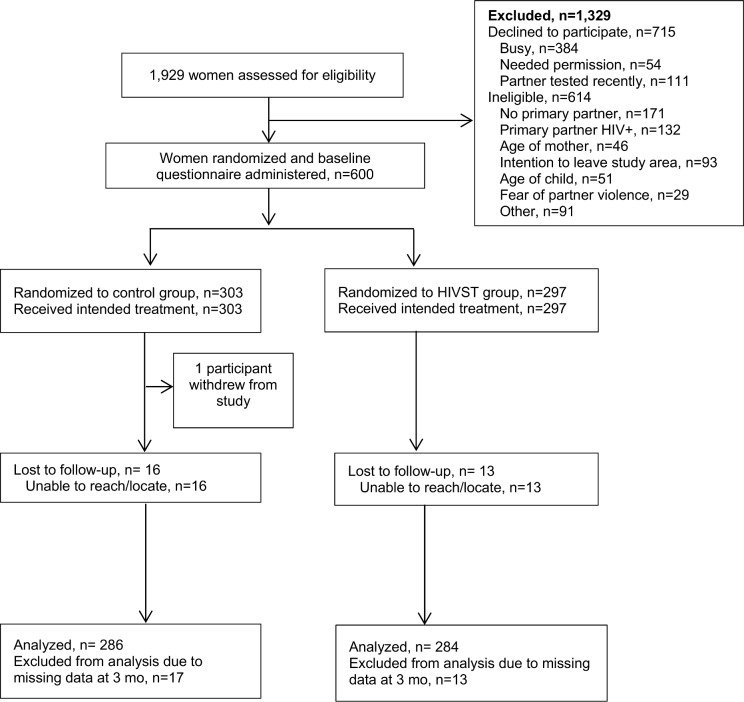
Assessment of eligibility, randomization, and follow-up. HIVST, HIV self-testing.

Between June 11, 2015, and October 16, 2015, a total of 1,929 women were screened for participation. Among those, 614 (32%) were determined to be ineligible, 715 declined to participate (37%), and 600 (31%) were enrolled and randomized (Fig 1). Reasons for ineligibility included no primary partner (28%), partner HIV-positive (22%), intention of leaving study area during follow-up period (15%), age of participant (8%), age of child (8%), and fear of IPV due to discussing HIV testing with partner (5%). Common reasons for refusal included women reporting they were “in a hurry” or “too busy” (384/715, 53.7%), needing permission from partner to enroll in a study (54/715, 7.6%), and reporting their partner had tested recently and therefore did not have interest in participating in the study (111/715, 15.5%). Follow-up interviews were conducted until January 15, 2016. One person from the comparison group withdrew from the study during the follow-up period. Of the 600 participants who were enrolled, follow-up was completed for 570 (95%), 286 (94.4%) in the comparison group and 284 (95.6%) in the HIVST group.

### Participant Characteristics

Participants in the two study groups had largely similar characteristics at baseline (Table 1). Their mean age was 24 y, and the vast majority were married. Median monthly earnings was US$0 since the majority did not report any engagement in income-earning activities during or after pregnancy. Participants’ self-reported sexual behavior and their reports of their partner’s HIV testing history were similar in both groups (Table 2). Nearly 4% of all participants self-reported being HIV-positive. The majority of participants reported that their partner had tested for HIV in the past 12 mo (56%), and only a small percentage of participants (14%) had heard of HIVST prior to the study. Nearly 30% of participants reported experiencing IPV in the past 12 mo.

**Table 1:** Baseline characteristics of study participants.

Characteristic	Comparison Group (*n =* 286)	HIV Self-Testing Group (*n =* 284)	Total (*n =* 570)
**Age (years), mean (SD)**	24.2 (4.3)	24.2 (4.5)	24.2 (4.4)
**Monthly earnings (US dollars), median (IQR)**	0 (0–30)	0 (0–40)	0 (0–36)
**Ethnic group**			
Luo	221 (77)	219 (77)	440 (77)
Luhya	33 (12)	43 (15)	76 (13)
Other	32 (11)	22 (8)	54 (9)
**Education**			
Some or completed primary	138 (48)	143 (50)	281 (49)
Some secondary	133 (47)	120 (42)	253 (44)
Completed secondary or greater	15 (5)	21 (7)	36 (6)
**Married**	266 (93)	266 (94)	532 (93)
**Occupation**			
Non-manual	74 (26)	83 (29)	157 (28)
Manual	19 (7)	28 (10)	47 (8)
Housewife/unemployed	193 (67)	173 (61)	366 (64)

For all variables frequencies are presented, with percentages in parentheses, except where otherwise noted.

IQR, interquartile range; SD, standard deviation.

**Table 2:** Self-reported sexual behavior and HIV testing history.

Behavior or HIV Testing History	Comparison Group (*n =* 286)	HIV Self-Testing Group (*n =* 284)	Total, (*n =* 570)
**Age at first intercourse (years), mean (SD)**	17.7 (2.8)	17.9 (2.5)	17.8 (2.7)
**Condom used during last sex**	54 (19)	46 (16)	100 (18)
**Had at least one other sexual partner in the past 12 mo**	4 (1)	5 (2)	9 (2)
**Number of times been tested for HIV in the past 12 mo, mean (SD)**	2.8 (1.4)	2.8 (1.5)	2.8 (1.4)
**Self-reported HIV-positive**	10 (3.5)	13 (4.6)	23 (4.1)
**Heard of HIV self-testing prior to study**	39 (14)	41 (14)	80 (14)
**Primary partner ever tested for HIV**			
Yes	220 (77)	216 (76)	436 (76)
No	19 (7)	21 (7)	40 (7)
Don’t know	47 (16)	47 (17)	94 (16)
**Primary partner tested for HIV in the past 12 mo**			
Yes	173 (60)	149 (52)	322 (56)
No	35 (12)	42 (15)	77 (14)
Don’t know	78 (27)	93 (33)	171 (30)
**Know partner’s status**	192 (67)	194 (68)	386 (68)
**Experienced intimate partner violence in the past 12 mo**	76 (27)	78 (27)	154 (27)

For all variables frequencies are presented, with percentages in parentheses, except where otherwise noted.

SD, standard deviation.

### Male Partner Testing

Male partner testing within 3 mo of enrollment was higher in the HIVST group (258/284, 90.8%) than the comparison group (148/286, 51.7%), as shown in Table 3. The difference of 39.1% between the two groups was statistically significant (95% CI 32.4% to 45.8%, *p <* 0.001). Among participants in the HIVST group whose partners used a self-test, 76% and 17% reported that their partner found it “very easy” or “somewhat easy,” respectively, to use the self-test, while 6% reported that their partner found it “somewhat difficult” or “very difficult.” In the comparison group, 45% (67/148) of partners who tested were reported to have done so outside of the three study facilities.

**Table 3:** Effects of HIV self-testing intervention within 3 mo.

Outcome	Comparison Group, Number (Percent) (*n =* 286)	HIV Self-Testing Group, Number (Percent) (*n =* 284)	Absolute Difference, Percentage Points (95% CI)*	Risk Ratio (95% CI)**	*p*-Value*
**Primary outcome**					
Male partner HIV testing	148 (51.7)	258 (90.8)	39.1 (32.4 to 45.8)	1.76 (1.56–1.98)	<0.001
**Secondary outcomes**					
Discussed HIV testing with partner	276 (96.5)	271 (95.4)	−1.1 (−4.3 to 2.2)	0.99 (0.96–1.02)	0.512
Couples testing for HIV	95 (33.2)	214 (75.4)	42.1 (34.7 to 49.6)	2.27 (1.90–2.71)	<0.001
Couples testing conditional on partner HIV testing***	95 (64.2)	214 (82.9)	18.8 (9.8 to 27.8)	1.29 (1.13–1.48)	<0.001
Aware of partner’s HIV test result	145 (50.7)	255 (89.8)	39.1 (32.3 to 45.9)	1.77 (1.57–2.00)	<0.001
Aware of partner’s HIV test result conditional on partner HIV testing***	145 (98.0)	255 (98.8)	0.9 (−1.8 to 3.5)	1.01 (0.98–1.04)	0.519
Partner tested HIV-positive	4 (1.4)	8 (2.8)	1.4 (−0.9 to 3.8)	2.01 (0.61–6.62)	0.239

*Estimates and confidence intervals are marginal effects from unadjusted modified Poisson regression.

**Estimates and confidence intervals are risk ratios from unadjusted modified Poisson regression.

***Model includes the subset of participants whose partner tested for HIV.

### Secondary Outcomes

Over 95% of participants in both groups reported discussing HIV testing with their partner since enrollment, and there was no significant difference between the two groups (difference = −1.1%, 95% CI −4.3% to 2.2%, *p* = 0.512). Participants in the HIVST group were more likely to test as a couple than participants in the comparison group (difference = 42.1%, 95% CI 34.7% to 49.6%, *p <* 0.001). In addition, among participants whose partner tested for HIV during the follow-up period, couples testing was more likely in the HIVST group than the comparison group (difference = 18.8%, 95% CI 9.8% to 27.8%, *p <* 0.001).

At follow-up, participants in the HIVST group were more likely to know their partner’s HIV status than those in the comparison group (difference = 39.1%, 95% CI 32.4% to 45.8%, *p <* 0.001). However, among participants whose partner tested for HIV during the follow-up period, participants’ awareness of their partner’s HIV status did not differ significantly between the two groups (difference = 0.9%, 95% CI −1.8% to 3.5%, *p <* 0.519), suggesting that the increase in awareness of partner HIV status in the HIVST group was driven by the greater likelihood of partner testing having occurred rather than a greater likelihood of becoming aware if a partner did get tested. Among participants whose partner tested for HIV, almost all were aware of their partner’s HIV test result (98.0% in comparison group, 98.8% in HIVST group). A small number of participants in both groups reported that their partner tested HIV-positive (1.4% in comparison group, 2.8% in HIVST group). Among the eight partners who tested positive in the HIVST group, two went for confirmatory testing, were confirmed positive, and were linked to care. Among the four partners who tested positive in the comparison group, three were reported to have sought HIV care at the time of the 3-mo interview. No participants in either group reported IPV due to HIV testing.

### Heterogeneity of Intervention Effectiveness

Participants in the HIVST group reported higher partner testing rates than participants in the comparison group in all subgroups examined (Table 4). While partner testing was significantly more likely in the HIVST group than the comparison group in all three study sites, the HIVST intervention was more effective in promoting partner testing in the hospital setting as compared to the urban health clinic setting (*p <* 0.001). There was no difference in intervention effectiveness by partner testing status in the past 12 mo (*p* = 0.172). Similarly, we found no difference in intervention effectiveness between participants who had experienced IPV at baseline and those who had not (*p* = 0.111).

**Table 4:** Comparison of intervention effectiveness in participant subgroups.

Subgroup	HIV Testing Uptake, Number/Total Number (Percent)		Effect of Self-Testing		*p*-Value for Interaction**
	Comparison Group	HIV Self-Testing Group	Absolute Difference, Percentage Points (95% CI)*	*p*-Value for Subgroup*	
**Study site**					
Urban health clinic	80/120 (66.7)	117/129 (90.7)	24.0 (14.2 to 33.9)	<0.001	—
Hospital	47/122 (38.5)	97/105 (92.4)	53.9 (43.8 to 63.9)	<0.001	<0.001
Peri-urban health clinic	21/44 (47.7)	44/50 (88.0)	40.3 (22.9 to 57.7)	<0.001	0.093
**Partner tested for HIV in 12 mo prior to enrollment**					
Tested ≥1 time	102/173 (59.0)	142/149 (95.3)	36.3 (28.3 to 44.4)	<0.001	—
Did not test	16/35 (45.7)	37/42 (88.1)	42.4 (23.1 to 61.7)	<0.001	0.389
Do not know if tested	30/73 (38.5)	79/93 (84.9)	46.5 (33.5 to 59.5)	<0.001	0.057
**Participants reported intimate partner violence in past 12 mo at baseline**					
No	114/210 (54.3)	185/206 (89.8)	35.5 (27.6 to 43.4)	<0.001	—
Yes	34/76 (44.7)	73/78 (93.6)	48.9 (36.4 to 61.3)	<0.001	0.111

*Estimates and confidence intervals are marginal effects from a modified Poisson regression of outcome on study group for the subgroup described.

**p-Value for interaction coefficient between subgroup and first category (urban health clinic, tested ≥1 time in past 12 mo, and no IPV).

## Discussion

Provision of multiple self-tests to women led to secondary distribution of the self-tests to their male partners and ultimately achieved higher HIV testing among their male partners and higher couples testing than a more conventional approach of giving women invitation cards for their male partners to test at health facilities. In the group that received multiple self-tests, partner testing was reported by 91% of participants who were followed up, and 75% of participants followed up tested together with their partner. To our knowledge, this is the first randomized trial to test whether provision of multiple self-tests to women promotes partner and couples testing. In subgroup analyses, the intervention was more effective than the partner invitation approach even among women who reported a history of IPV at baseline and among women whose partners had not gone for HIV testing in the past 12 mo.

Male partner testing was nearly universal among women who received multiple self-tests. This striking result is consistent with findings from a pilot study we previously conducted in the study region, in which male partner testing was reported to have occurred for 91% of women seeking ANC and 86% of women receiving PNC [22]. The study results are also consistent with the high acceptability of HIVST that has been documented throughout SSA and elsewhere [15–17].

Uptake of partner testing and couples testing in the comparison group that received invitation cards for their male partner was largely similar to what has been reported in two other recent studies. One study conducted in the same region of Kenya reported that couples testing occurred among 36% of pregnant women who received clinic invitation cards for their partner [26]. Further, a study conducted among HIV-positive pregnant women in Malawi reported that couples testing occurred among 52% of women who received invitation cards for their partner [27]. The similarity in male partner and couples testing levels in the comparison group of our study with those reported in these other studies of the partner invitation approach provide further support for the validity of the self-reported measures obtained in our study. In addition, it is notable that the couples testing rate in the HIVST group of our study was similar to or exceeded the rates achieved by the interventions tested in the two other studies: home visits and invitations followed by home tracing. While formal cost-effectiveness analyses are necessary, it is plausible that interventions relying on secondary distribution of self-tests would ultimately require fewer resources in total and therefore would have greater sustainability.

While prior HIV testing in this urban and peri-urban study setting was fairly high, we found no difference in the effectiveness of the HIVST intervention based on whether partners had tested for HIV in the past 12 mo. This result is encouraging since it suggests that the strategy of giving multiple self-tests to women can effectively increase access to HIV testing in hard-to-reach populations such as men who do not test regularly, and perhaps more generally in settings where testing rates are not as high as they were in our study setting. In addition, the large differences in partner testing between the HIVST and comparison groups was observed in all population subgroups, which suggests broader applicability of this intervention to various subgroups of pregnant and postpartum women.

From a policy standpoint, providing self-tests to women in clinic settings has substantial appeal not only because it promotes male partner testing but also because it helps women learn their partner’s HIV status. The intervention’s feasibility is enhanced by the fact that pregnant and postpartum women represent an easier-to-reach segment of the population by virtue of their higher utilization of health services. Couples testing, which is recommended by the World Health Organization and the Kenyan Ministry of Health, is another important benefit of the intervention. Individuals who test as a couple and mutually disclose their HIV status are more likely than those testing alone to adopt a range of HIV prevention and care behaviors [5]. Despite these benefits, only 37.2% of people who have tested for HIV in Kenya reported ever testing together with a sexual partner [28]. Notably, the uptake of couples testing observed among women given multiple self-tests in this study (75%) was higher than the uptake reported in the recent pilot study we conducted in the study area, in which women receiving ANC and PPC tested as couples 47% and 58% of the time, respectively [22].

This study has several limitations that warrant discussion. First, we relied on self-reported data for the main outcomes. This is a common limitation in many studies involving HIVST due to the private manner in which self-tests are meant to be used. Despite the potential for self-reporting to be associated with reporting bias, we believe reporting bias was minimal given the above-mentioned consistency of our results for partner testing in both study groups with other studies conducted in SSA [15,18,22,26,27] and given the lack of material incentives tied to participants’ responses. In addition, any bias in reporting of testing uptake is unlikely to be differential by study group. Male partners in the comparison group were able to test at multiple facilities in the study area, and it was as difficult in practice to verify their clinic-based testing as it was to verify self-test usage by partners in the HIVST group. These factors are likely to strengthen the validity of comparing self-reported partner testing in the two study groups. Since objective verification of self-test use will remain a challenge, there is a need for larger-scale studies that examine downstream outcomes such as the proportion of partners linking to HIV prevention and treatment. Second, our study did not include women who knew their partner was HIV-positive because we believed that a partner testing intervention would have little additional benefit to them. This feature of the study design, coupled with high rates of HIV testing in the urban and peri-urban study setting [29], likely led to relatively few HIV-positive partners being identified in this study. This limited our ability to make statistical inferences with respect to confirmatory testing and linkage to care. More research is needed to rigorously assess levels of confirmatory testing and linkage to care following HIVST, as well as to understand the decision-making process of whether or not to seek these services.

Finally, the third limitation stems from the fact that roughly one-third of women seeking ANC or PPC declined to participate in the study, and some were ineligible because they reported a fear that violence would result from offering a self-test to their partner. Among women declining participation, the most commonly reported reason was a lack of adequate time to enroll in the study, but other reasons such as a lack of interest in partner testing likely played a role. While these two reasons for declining to participate in the study do not impact the internal validity of the study results, they do limit the generalizability of the findings to all pregnant and postpartum women. Refusal also reinforces the feasibility and safety of offering multiple self-tests because women demonstrated considerable agency and ability to decide themselves whether to accept self-tests and offer them to their partner. Prior work has documented the high acceptability of this intervention among women receiving multiple self-tests [22], and ongoing qualitative research with women receiving multiple self-tests shows that women have a strong sense of agency when deciding whether to offer self-tests to others and appreciate the opportunity to learn their partner’s status. Additional qualitative research will provide insights and lessons for wider implementation. Given the novelty of HIVST and this particular strategy for promoting partner testing (i.e., secondary distribution of self-tests by women receiving ANC and PPC), it is also likely that the broader acceptability of secondary distribution strategies will grow as HIVST becomes more common. Additional research is necessary to assess the effectiveness of the intervention in other populations and settings outside western Kenya. However, to the extent that men experience similar barriers to clinic-based HIV testing elsewhere, the results from this study could be applicable to other settings and populations.

One concern about providing multiple self-tests to women for distribution to partners has been the possibility of IPV. Despite women reporting high rates of IPV in the past 12 mo at baseline (27%), it is noteworthy that the intervention was highly effective even among women who reported a history of IPV at baseline, and there were no cases of IPV due to HIV testing reported in either study group during the follow-up period. Few male partners had a reactive self-test result in the study, which may have contributed to the lack of reported IPV due to testing. However, prior research we have conducted with women receiving multiple self-tests—including female sex workers who identified a greater proportion of HIV-positive partners than ANC or PPC women in our study—also suggests IPV is rare [22]. The fact that there were no cases of IPV also suggests that women have the agency and discretion to decide whether to accept self-tests and whether to offer self-tests to their partner.

This study provides key insights on a strategy—secondary distribution of self-tests to sexual partners—that may become common in many populations in SSA and elsewhere as HIV self-tests become more widely available, whether formally endorsed or not. For example, the feasibility of this approach is also being explored among key populations such as men who have sex with men [30,31]. The promising results from this study suggest that secondary distribution of self-tests warrants further consideration as countries develop HIVST policies and seek new ways to promote partner testing. Implementing this intervention at scale is feasible as the primary requirements are that clinic staff be trained on how to explain self-test use and to offer self-tests to women. However, there are potential challenges to programmatic implementation of the intervention, such as ensuring adequate counseling when self-tests are offered to women, making counseling available post-test, and including interventions to achieve high linkage to appropriate services. Ongoing and planned implementation research will assess these issues and further develop strategies for maximizing the potential for HIVST in achieving HIV prevention and care objectives.

## Supporting Information

S1 Table Outcome and intimate partner violence questions. (DOCX)

S1 Text Study protocol. (DOCX)

S2 Text CONSORT checklist. (DOC)

## Abbreviations

ANC: antenatal care
HIVST: HIV self-testing
IPV: intimate partner violence
PPC: postpartum care
SSA: sub-Saharan Africa

## References

[1] UNAIDS. 90-90-90: an ambitious treatment target to help end the AIDS epidemic. Geneva: UNAIDS; 2014.

[2] Suthar AB, Ford N, Bachanas PJ, Wong VJ, Rajan JS, Saltzman AK, et al Towards universal voluntary HIV testing and counselling: a systematic review and meta-analysis of community-based approaches. PLoS Med. 2013;10(8):e1001496 Epub 2013/08/24. PubMed Central PMCID: PMC3742447. 10.1371/journal.pmed.1001496 23966838 PMC3742447

[3] Sharma M, Ying R, Tarr G, Barnabas R. Systematic review and meta-analysis of community and facility-based HIV testing to address linkage to care gaps in sub-Saharan Africa. Nature. 2015;528(7580):S77–85. 10.1038/nature16044 26633769 PMC4778960

[4] Gelmon L, Kenya P, Oguya F, Cheluget B, Haile G. Kenya HIV prevention response and modes of transmission analysis Nairobi: Kenya National AIDS Control Council; 2009.

[5] Allen S, Meinzen-Derr J, Kautzman M, Zulu I, Trask S, Fideli U, et al Sexual behavior of HIV discordant couples after HIV counseling and testing. AIDS. 2003;17(5):733–40. 10.1097/01.aids.0000050867.71999.ed 12646797

[6] Cohen MS, Chen YQ, McCauley M, Gamble T, Hosseinipour MC, Kumarasamy N, et al Prevention of HIV-1 infection with early antiretroviral therapy. N Engl J Med. 2011;365(6):493–505. 10.1056/NEJMoa1105243 21767103 PMC3200068

[7] Aluisio A, Richardson BA, Bosire R, John-Stewart G, Mbori-Ngacha D, Farquhar C. Male antenatal attendance and HIV testing are associated with decreased infant hiv infection and increased HIV free survival. J Acquir Immune Defic Syndr. 2011;56(1):76–82. 10.1097/QAI.0b013e3181fdb4c4 21084999 PMC3005193

[8] Drake AL, Wagner A, Richardson B, John-Stewart G. Incident HIV during pregnancy and postpartum and risk of mother-to-child HIV transmission: a systematic review and meta-analysis. PLoS Med. 2014;11(2):e1001608 10.1371/journal.pmed.1001608 24586123 PMC3934828

[9] Medley A, Baggaley R, Bachanas P, Cohen M, Shaffer N, Lo Y-R. Maximizing the impact of HIV prevention efforts: interventions for couples. AIDS Care. 2013;25(12):1569–80. 10.1080/09540121.2013.793269 23656251 PMC4664148

[10] World Health Organization. Guidance on couples HIV testing and counselling including antiretroviral therapy for treatment and prevention in serodiscordant couples: recommendations for a public health approach Geneva: World Health Organization; 2012. 23700649

[11] Farquhar C, Kiarie JN, Richardson BA, Kabura MN, John FN, Nduati RW, et al Antenatal couple counseling increases uptake of interventions to prevent HIV-1 transmission. J Acquir Immune Defic Syndr. 2004;37(5):1620–6. Epub 2004/12/04. PubMed Central PMCID: PMCPmc3384734. 15577420 10.1097/00126334-200412150-00016 PMC3384734

[12] Msuya SE, Mbizvo EM, Hussain A, Uriyo J, Sam NE, Stray-Pedersen B. Low male partner participation in antenatal HIV counselling and testing in northern Tanzania: implications for preventive programs. AIDS Care. 2008;20(6):700–9. Epub 2008/06/26. 10.1080/09540120701687059 18576172

[13] Obermeyer CM, Osborn M. The utilization of testing and counseling for HIV: a review of the social and behavioral evidence. Am J Public Health. 2007;97(10):1762–74. Epub 2007/09/01. PubMed Central PMCID: PMCPMC1994175. 10.2105/AJPH.2006.096263 17761565 PMC1994175

[14] Hutchinson AB, Corbie-Smith G, Thomas SB, Mohanan S, del Rio C. Understanding the patient’s perspective on rapid and routine HIV testing in an inner-city urgent care center. AIDS Educ Prev. 2004;16(2):101–14. Epub 2004/05/12. 15134119 10.1521/aeap.16.2.101.29394

[15] Choko AT, Desmond N, Webb EL, Chavula K, Napierala-Mavedzenge S, Gaydos CA, et al The uptake and accuracy of oral kits for HIV self-testing in high hiv prevalence setting: a cross-sectional feasibility study in Blantyre, Malawi. PLoS Med. 2011;8(10):e1001102 10.1371/journal.pmed.1001102 21990966 PMC3186813

[16] Figueroa C, Johnson C, Verster A, Baggaley R. Attitudes and acceptability on HIV self-testing among key populations: a literature review. AIDS Behav. 2015;19(11):1949–65. PubMed Central PMCID: PMC4598350. 10.1007/s10461-015-1097-8 26054390 PMC4598350

[17] Napierala Mavedzenge S, Baggaley R, Corbett EL. A review of self-testing for HIV: research and policy priorities in a new era of HIV prevention. Clin Infect Dis. 2013;57(1):126–38. 10.1093/cid/cit156 23487385 PMC3669524

[18] Choko AT, MacPherson P, Webb EL, Willey BA, Feasy H, Sambakunsi R, et al Uptake, accuracy, safety, and linkage into care over two years of promoting annual self-testing for HIV in Blantyre, Malawi: a community-based prospective study. PLoS Med. 2015;12(9):e1001873 Epub 2015/09/09. PubMed Central PMCID: PMCPmc4562710. 10.1371/journal.pmed.1001873 26348035 PMC4562710

[19] National AIDS and STI Control Programme. Guidelines for HIV testing services in Kenya Nairobi: National AIDS and STI Control Programme; 2015.

[20] World Health Organization. Consolidated guidelines on HIV testing services Geneva: World Health Organization; 2015. 26378328

[21] National AIDS and STI Control Programme, Ministry of Public Health and Sanitation. Guidelines for HIV testing and counselling in Kenya Nairobi: National AIDS and STI Control Programme; 2010.

[22] Thirumurthy H, Masters S, Napierala Mavedzenge S, Maman S, Omanga E, Agot A. Promoting male partner HIV testing and safer sexual decision making through secondary distribution of self-tests by HIV-negative female sex workers and women receiving antenatal and post-partum care in Kenya: a cohort study. Lancet HIV. 2016;3(6):e266–74. 10.1016/S2352-3018(16)00041-2 27240789 PMC5488644

[23] National AIDS and STI Control Programme. Kenya HIV county profiles Nairobi: Ministry of Health; 2014.

[24] Zou G. A modified poisson regression approach to prospective studies with binary data. Am J Epidemiol. 2004;159(7):702–6. Epub 2004/03/23. 15033648 10.1093/aje/kwh090

[25] Kenya National Bureau of Statistics, ICF Macro. Kenya Demographic and Health Survey 2008–09. Calverton (Maryland): ICF Macro; 2010.

[26] Osoti AO, John-Stewart G, Kiarie J, Richardson B, Kinuthia J, Krakowiak D, et al Home visits during pregnancy enhance male partner HIV counselling and testing in Kenya: a randomized clinical trial. AIDS. 2014;28(1):95–103. Epub 2013/08/15. PubMed Central PMCID: PMCPmc4337402. 10.1097/QAD.0000000000000023 23942059 PMC4337402

[27] Rosenberg NE, Mtande TK, Saidi F, Stanley C, Jere E, Paile L, et al Recruiting male partners for couple HIV testing and counselling in Malawi’s option B+ programme: an unblinded randomised controlled trial. Lancet HIV. 2015;2(11):e483–91. 10.1016/S2352-3018(15)00182-4 26520928 PMC4656790

[28] Ng’ang’a A, Waruiru W, Ngare C, Ssempijja V, Gachuki T, Njoroge I, et al The status of HIV testing and counseling in Kenya: results from a nationally representative population-based survey. J Acquir Immune Defic Syndr. 2014;66(Suppl 1):S27–36. Epub 2014/04/16. 10.1097/QAI.0000000000000102 24732818 PMC4786172

[29] Kimanga DO, Ogola S, Umuro M, Ng’ang’a A, Kimondo L, Murithi P, et al Prevalence and incidence of HIV infection, trends, and risk factors among persons aged 15–64 years in Kenya: results from a nationally representative study. J Acquir Immune Defic Syndr. 2014;66(Suppl 1):S13–26. PubMed Central PMCID: PMCPMC4794992. 10.1097/QAI.0000000000000124 24445338 PMC4794992

[30] Carballo-Dieguez A, Frasca T, Balan I, Ibitoye M, Dolezal C. Use of a rapid HIV home test prevents HIV exposure in a high risk sample of men who have sex with men. AIDS Behav. 2012;16(7):1753–60. Epub 2012/08/16. PubMed Central PMCID: PMC3458207. 10.1007/s10461-012-0274-2 22893194 PMC3458207

[31] Carballo-Dieguez A, Frasca T, Dolezal C, Balan I. Will gay and bisexually active men at high risk of infection use over-the-counter rapid HIV tests to screen sexual partners? J Sex Res. 2012;49(4):379–87. Epub 2012/02/02. 10.1080/00224499.2011.647117 22293029 PMC3600862

