# Supplementary material for: Promoting Partner Testing and Couples Testing through Secondary Distribution of HIV Self-Tests: A Randomized Clinical Trial
Source: PLoS Med. 2016 Nov 8;13(11):e1002166. doi: 10.1371/journal.pmed.1002166 (PMC5100966; doi:10.1371/journal.pmed.1002166)
Supplement: S1 Table — (DOCX) [file pmed.1002166.s001.docx]

Supplementary Appendix

Baseline IPV questions

| Section D. Gender-Based Violence | | | |
| --- | --- | --- | --- |
| Interviewer: “I always ask the following questions because some people are in relationships where they don’t feel safe and this affects their health.” | | | |
| 1 | If you told your partner that you came here for health services today, would he react angrily or negatively? | 1 YES  2 NO  98 Don’t Know  99 Refused |  |
|  | In the past 12 months, has your partner… |  |  |
| 2 | Pushed, grabbed, slapped, choked, hit or kicked you? | 1 YES  2 NO  98 Don’t Know  99 Refused |  |
| 3 | Threatened to hurt you, your children or someone close to you? | 1 YES  2 NO  98 Don’t Know  99 Refused |  |
| 4 | Insulted you or made you feel bad about yourself? | 1 YES  2 NO  98 Don’t Know  99 Refused |  |
| 5 | Taken away money or resources that you/your children need to survive? | 1 YES  2 NO  98 Don’t Know  99 Refused |  |
| 6 | Sent you back to your family home? | 1 YES  2 NO  98 Don’t Know  99 Refused |  |
| 7 | Forced you to have sex when you did not want to? | 1 YES  2 NO  98 Don’t Know  99 Refused |  |
| 8 | Has your partner tried to get you pregnant when you didn’t want to be? | 1 YES  2 NO  98 Don’t Know  99 Refused |  |
| 9 | If you wanted to use a condom or another family planning method, would you be afraid to ask your partner? | 1 YES  2 NO  98 Don’t Know  99 Refused |  |
| 10 | Are you worried that your partner will be angry and/or hurt you if he finds out you were tested for HIV? | 1 YES  2 NO  98 Don’t Know  99 Refused |  |
| 11 | Do you feel unsafe returning to your home today? | 1 YES  2 NO  98 Don’t Know  99 Refused |  |

Follow-up IPV questions

| **Section E.** IPV Screening | | | | |
| --- | --- | --- | --- | --- |
| NO. | **QUESTIONS** | | **CODING CATEGORIES** |  |
|  | | In the past 1 (2, 3) months, has your partner… | **[time depends on when follow up interview occurs]** |  |
| 1 | | Pushed, grabbed, slapped, choked, hit or kicked you? | **1 YES**  **2 NO**  **98 Don’t Know**  **99 Refused** |  |
| 2 | | Threatened to hurt you, your children or someone close to you? | **1 YES**  **2 NO**  **98 Don’t Know**  **99 Refused** |  |
| 3 | | Insulted you or made you feel bad about yourself? | **1 YES**  **2 NO**  **98 Don’t Know**  **99 Refused** |  |
| 4 | | Taken away money or resources that you/your children need to survive? | **1 YES**  **2 NO**  **98 Don’t Know**  **99 Refused** |  |
| 5 | | Sent you back to your family home? | **1 YES**  **2 NO**  **98 Don’t Know**  **99 Refused** |  |
| 6 | | Forced you to have sex when you did not want to? | **1 YES**  **2 NO**  **98 Don’t Know**  **99 Refused** |  |
| 7 | | Has your partner tried to get you pregnant when you didn’t want to be? | **1 YES**  **2 NO**  **98 Don’t Know**  **99 Refused** |  |
| 8a | | [If yes to any of the above GBV questions] Did this happen as a result of discussing HIV testing with your partner? | **1 YES**  **2 NO**  **98 Don’t Know**  **99 Refused** |  |
| 8b | | Do you think any of these things were the result of having offered an HIV self-test to your partner? | **1 YES**  **2 NO**  **98 Don’t Know**  **99 Refused** | If offered ST |
| 9 | | Do you feel unsafe returning to your home today? | **1 YES**  **2 NO**  **98 Don’t Know**  **99 Refused** |  |

Outcomes questions

For clinic-based testing group

| Section C1. HIV Testing in control group | | | |
| --- | --- | --- | --- |
| **NO.** | **QUESTIONS** | **CODING CATEGORIES** | **NOTES** |
| 1 | Since enrolling in this study, have you discussed HIV testing with your partner? | 1 Yes  2 No🡪C6  98 Don’t know🡪C6  99 Refused to answer🡪C6 |  |
| 6 | Has your partner had an HIV test since you were enrolled in the study? | 1 Yes  2 No🡪C20  98 Don’t know🡪C20  99 Refused to answer🡪C20 |  |
| 9 | When your partner was tested, did he test individually (not with you) or together with you? | 1 Individually (Partner testing)  2 Together with you (Couples testing)  3 Other (describe…)  98 Don’t know  99 Refused to answer | Ask if C6=1 |
| 10 | Do you know the results of your partner’s HIV test? | 1 Yes  2 No🡪C13  98 Don’t know🡪C13  99 Refused to answer | Ask if C6=1 |
| 11 | What was your partner’s test result?  Interviewer: Remind participant that they do not have to answer this question if uncomfortable. If she says that she knows but doesn’t want to share – this is code 99. If the person tested but did not share the result with them so they don’t know, this is code 98 | 1 HIV-positive  2 HIV-negative  3 Indeterminate  98 Don’t know  99 Refused to answer | Ask if C10=1 |
| 12 | Did your partner go to a clinic or health facility for HIV care? | 1 Yes  2 No  98 Don’t know  99 Refused to answer | Ask if C11=1 |

For self-testing group

| Section C2. HIV Testing in intervention group (administer only if A3=1) | | | |
| --- | --- | --- | --- |
| **NO.** | **QUESTIONS** | **CODING CATEGORIES** | **NOTES** |
| 1 | Since enrolling in this study, have you discussed HIV testing with your partner? | 1 Yes  2 No🡪C6A  98 Don’t know  99 Refused to answer | C1=2🡪C6A |
| 6A | Has your partner had an HIV test since you were enrolled in the study?  Interviewer: probe if No and make sure that response applies to all forms of HIV testing (clinic-based or self-testing) | 1 Yes  2 No🡪C6B  98 Don’t know🡪C6B  99 Refused to answer🡪C6B |  |
| 6B | Did you give your partner an HIV self-test since you were enrolled in the study? | 1 Yes  2 No🡪C18  98 Don’t know🡪C18  99 Refused to answer🡪C18 |  |
| 6C | Did your partner use the HIV self-test that you gave him?  *Interviewer*: if yes, probe if participant was present or not when the partner used the test. Remind the participant that we are now talking about USAGE of self-test | 1 Yes – I was present when he used the self-test  2 Yes – I was told that the self-test was used  3 No – he did not use the self-test  98 Don’t know  99 Refused to answer |  |
| 9a | When your partner used the self-test, did you also use a self-test at the same time? | 1 Yes  2 No  98 Don’t know  99 Refused | Ask if C6c=1 or 2 |
| 9b | When your partner was tested, did he test individually (not with you) or together with you? | 1 Individually (Partner testing)  2 Together with you (Couples testing)  3 Other (describe…)  98 Don’t know  99 Refused to answer | Ask if C6a=1 & [is control or 6b=2 or 6c=3] |
| 10 | Do you know the results of your partner’s HIV test? | 1 Yes  2 No  98 Don’t know  99 Refused to answer | Ask if C6a=1 |
| 11 | What was your partner’s test result?  *Interviewer*: Remind participant that they do not have to answer this question if uncomfortable. If she says that she knows but doesn’t want to share – this is code 99. If the person tested but did not share the result with them so they don’t know, this is code 98 | 1 HIV-positive  2 HIV-negative  3 Indeterminate  98 Don’t know  99 Refused to answer | Ask if C10=1 |
| 12 | Did your partner go to a clinic or health facility in order to confirm the result that was obtained from the self-test (confirmatory testing)? | 1 Yes  2 No  98 Don’t know  99 Refused to answer | Ask if C6c=1 or 2 |
| 13 | Did your partner go to a clinic or health facility for HIV care? | 1 Yes  2 No  98 Don’t know  99 Refused to answer | Ask if C12=1 |
