# Supplementary material for: Promoting Partner Testing and Couples Testing through Secondary Distribution of HIV Self-Tests: A Randomized Clinical Trial
Source: PLoS Med. 2016 Nov 8;13(11):e1002166. doi: 10.1371/journal.pmed.1002166 (PMC5100966; doi:10.1371/journal.pmed.1002166)
Supplement: S1 Text — (DOCX) [file pmed.1002166.s002.docx]

**The use of HIV self-tests to promote partner and couples testing:a randomized trial**

**UNC / IRDO**

**INVESTIGATORS AND INSTITUTIONAL AFFILIATIONS**

| Co-Principal Investigator: | Harsha Thirumurthy, PhD Assistant Professor of Health Economics Department of Health Policy and Management  Gillings School of Global Public Health  Carolina Population Center  University of North Carolina at Chapel Hill, Chapel Hill, NC, USA |
| --- | --- |
| Co-Principal Investigator: | KawangoAgot, PhD, MPH  Director  Impact Research and Development Organization, Kisumu, Kenya |
| Co-Investigators: | Eunice Omanga, DrPH  Head of Research  Impact Research and Development Organization, Kisumu, Kenya |
| Immaculate Akello, B.A, MPhil  Study Coordinator  Impact Research and Development Organization, Kisumu, Kenya |  |
| Co-Investigators: | Suzanne Maman, PhD  Associate Professor  Department of Health Behavior  University of North Carolina at Chapel Hill, Chapel Hill, NC, USA |
| Sue Napierala Mavedzenge, PhD, MPH  Research Epidemiologist  RTI International, Research Triangle Park, NC, USA |  |
| Arianna Zanolini, PhD  Post-Doctoral Fellow  Center for Infectious Diseases and Research in Zambia  University of North Carolina at Chapel Hill, Chapel Hill, NC, USA |  |
|  | Kate Murray, MPH  Study Coordinator  Carolina Population Center  University of North Carolina at Chapel Hill, Chapel Hill, NC, USA |

**Proposed Duration**: 13 months (October 2014–December 2015)

**Funding**: International Initiative for Impact Evaluation (3ie), Thematic Window 2**TABLE OF CONTENTS**

[LIST OF ACRONYMS 6](#_Toc391021482)

[1.0 INTRODUCTION/BACKGROUND 7](#_Toc391021484)

[2.0 JUSTIFICATION 9](#_Toc391021485)

[3.0 STUDY OBJECTIVES AND HYPOTHESES 10](#_Toc391021486)

[4.0 STUDY DESIGN 12](#_Toc391021487)

[4.1 Overview of study design 12](#_Toc391021488)

[4.2 Study site 12](#_Toc391021489)

[4.3 Study population 13](#_Toc391021490)

[4.3.1 Inclusion criteria 13](#_Toc391021491)

[4.3.2 Exclusion criteria 13](#_Toc391021492)

[4.4 Sampling 13](#_Toc391021493)

[4.5 Procedures 13](#_Toc391021494)

[4.6 Study withdrawal for participants 16](#_Toc391021495)

[4.7 Adverse events 16](#_Toc391021496)

[5.0 ETHICAL CONSIDERATIONS 18](#_Toc391021497)

[5.1 Informed Consent 18](#_Toc391021498)

[5.2 Ethical Approval 18](#_Toc391021499)

[5.3 Protecting privacy and confidentiality 18](#_Toc391021500)

[5.4 Potential risks of proposed research to study participants 18](#_Toc391021501)

[5.5 Potential benefits of proposed research to study participants and others 19](#_Toc391021502)

[6.1 Data storage 19](#_Toc391021503)

[6.2 Data management 19](#_Toc391021504)

[7.0 STUDY TIMELINE 20](#_Toc391021505)

[8.0 EXPECTED APPLICATION OF THE RESULTS 21](#_Toc391021506)

[11.0 ROLES AND RESPONSIBILITIES 23](#_Toc391021507)

[12.0 REFERENCES](#_Toc391021509) 20

ABSTRACT

This randomized controlled trial will explore whether the provision of oral HIV self-test kits to womencan increase partner testing. The study will recruit adult women from antenatal care and post-partum clinics in Kisumu. Women who are randomized to the intervention group will be provided with multiple oral self-tests as well as inform them about how to use the tests and where to seek care if necessary. These women will be encouraged to give the self-teststo their sexual partners and encourage them to take the self-test. Women who are randomized to the control group will be offered the standard counseling to refer their partners for clinic-based HIV counseling and testing. Over a 3 month period, we will obtain information from study participants on how many sexual partners they offered the tests to, the receptivity of their sexual partners to using self-tests, and the incidence of any adverse events. We also compare partner testing rates in intervention and control groups.

# LIST OF ACRONYMS

ANC Antenatal Care

HIV Human Immunodeficiency Virus

HTC HIV testing and counseling

IDI In-depth Interview

IPV Intimate Partner Violence

IRDO Impact Research & Development Organization

KEMRI Kenya Medical Research Institute

LHC Lumumba Health Centre

ODK Open Data Kit

PPC Postpartum Care

RA Research Assistant

SOP Standard Operating Procedures

UNC-CH University of North Carolina at Chapel Hill

VCT Voluntary Testing & Counseling

|  |  |
| --- | --- |

# INTRODUCTION/BACKGROUND

Increasing the uptake of HIV testing and counseling (HTC) in sub-Saharan Africa is essential for improving the effectiveness of HIV treatment and preventing new HIV infections. Although Kenya has nearly met its target of 80% coverage of HTC among adults, uptake of HTC among men, uptake of repeat testing, and knowledge of HIV status among HIV-infected persons remains low[1, 2]. Whereas early diagnosis and treatment reduces transmission[3-5], improves survival and reduces long-term morbidity[4, 6, 7], late diagnosis is more common and a major contributor to high rates of early mortality in African HIV care programs[8, 9]. Low HTC uptake therefore limits the impact of combination HIV prevention, including treatment as prevention strategies.Existing efforts to encourage women to refer their male partners for HTC and thereby address the male testing gap have had limited success. Moreover, despite the benefits of couples testing – greater likelihood of mutual HIV status disclosure and adoption of antiretroviral therapy for HIV prevention – the majority of HIV testing takes place alone and not with sexual partners. Innovative use of HIV self-tests provides an opportunity to address the challenges of low uptake of HIV testing among men and among couples.

Since adult men are significantly less likely to seek HTC than women, finding novel ways to close the male testing gap is a major priority. Women in Kenya access HIV testing at higher rates than men, in large part due to the offer of routine HIV testing for pregnant women at antenatal clinics and generally higher healthcare utilization among women. Results from the Kenya AIDS Indicator Survey (KAIS) indicate that only 62.5% have ever tested for HIV and of those, only 63.9% had tested for HIV more than once (compared to 80.4% and 73.3% of women)[10]. These data along with other studies have pointed to the need for innovative HIV testing strategies for men [10]. One commonly used method for engaging more men in HTC has been to recommend to pregnant women that they ask their partners to accompany them for antenatal care [11]. But in practice this approach has not succeeded in increasing uptake of HTC by men in a significant manner. The proportion of Kenyan men accompanying female partners to antenatal appointments is low (16%) although encouragingly 95% of those accept HIV testing [12].

Couples testing also has important HIV prevention benefits and has been recommended for scale-up, but there has been limited success in increasing its uptake.Couples testing has been recommended by the World Health Organization’s guidelines along with support for mutual disclosure of HIV status and antiretroviral therapy (ART) for serodiscordant couples[11]. Foremost among these benefits is the possibility of better sexual decision-making (for example, marital and fertility decisions). Individuals in couples who test together and mutually disclose their HIV status are also more likely than those testing alone to adopt a range of HIV prevention behaviors. Among HIV-infected pregnant women in particular, couples testing can results in greater support for uptake and adherence to prevention of mother-to-child transmission (PMTCT) interventions. Among serodiscordant couples, the benefits include a greater likelihood of ART uptake and adherence to improve the HIV-infected person’s health and reduce HIV transmission risk; as well as the possibility (in some cases) of the uninfected individual accessing pre-exposure prophylaxis (PrEP) to prevent HIV acquisition. Despite these benefits,according to the KAIS data only 37.2% of testers had reported ever testing together with a sexual partner [10].

Low uptake of HIV testing among men and among couples in Kenya is concerning in light of data indicating that 4 in 10 new HIV infections occur within stable heterosexual partnerships and the majority of persons in serodiscordant relationships are unaware of their HIV status [13].Although HTC is available free-of-charge in most places, for many individuals and couples the barriers to testing include stigma, fear of prognosis, lack of awareness of HIV risk, inconvenience, fear of disclosure, transportation costs, opportunity costs such as time off from work, and behavioral factors such as a tendency to put off behaviors with immediate costs and delayed benefits [14, 15].

Oral fluid-based HIV self-testing is a promising biomedical technology that has the potential to overcome many barriers to HIV testing.Self-tests, which enable individuals to test themselves and their partners for HIV privately, may first and foremost help to increase the number of people who are aware of their own HIV status. Self-tests also provide individuals with an opportunity to initiate testing with their sexual partners, a possibility that could have significant implications for HIV prevention.With self-testing, individuals collect their own sample and perform a simple, rapid HIV antibody test in the absence of a provider. It offers increased convenience, privacy, and autonomy, and has the potential to increase normalization of regular testing. With the development of simple, oral fluid-based tests, regulated self-testing has recently been adopted in Kenya [16].

Existing research shows a high level of acceptability and demand for self-testing. Importantly for our intervention, men in Kenya have shown considerable openness to self-testing [10, 17]. In studies from the US, Europe and Africa, 73% to 89% of people have reported interest in, preference for, or belief that self-testing should be made available[18-24]. Interest is typically highest among high-risk groups and those who have never tested for HIV [20-22]. In a community-based study in Malawi, 92% of participants opted for supervised self-testing over provider-delivered HTC, including a high proportion of first-time testers[25]. Data from six European countries indicated that 67% of self-testers would not have tested through provider-delivered HTC [26].These data suggest that HIV self-testing has the potential to reach new populations who are not comfortable accessing existing HTC services. Respondents from a range of studies report that self-testing would reduce or eliminate stigma around HIV testing, a formidable barrier, as well as increase convenience and confidentiality [18, 21, 22, 27, 28]. In terms of accuracy of self-testing, US emergency room patients achieved 100% accuracy, and in the study in Malawi accuracy was >99% [25]. The FDA-approved Oraquick In-home HIV test achieved 92% sensitivity in premarket self-testing in the US [29]. A recent study in Kenya found 92.9% sensitivity and 97.8% specificity [30].

A promising but under-explored use of self-testing is the provision of multiple self-tests to individuals who are then encouraged to distribute the self-tests in their sexual networks.Given the low uptake of HTC by men and by couples so far and the high risk of HIV infection among individuals in stable heterosexual partnerships, an especially promising use of self-testing could lie in the initiation of partner testing or couples testing by providing multiple self-tests to index individuals who are in stable partnerships [31]. So far there is limited evidence on the feasibility of a strategy that would provide multiple HIV self-tests to an individual and encourage them to use the tests to make more informed sexual decisions and better protect themselves from acquiring HIV. A small qualitative study in Malawi found that couples were able to use self-testing to facilitate mutual HIV-status disclosure, which further supports the feasibility of our intervention [32]. While such use of self-tests within partnerships in sub-Saharan Africa is highly innovative, there have been efforts to use self-tests in this way in pilot studies among men who have sex with men (MSM) in New York City [21, 33, 34]. MSM were able to use self-tests to screen potential sexual partners for HIV and decide whether to use condoms or avoid sex altogether when partners declined to test or when an HIV-positive result was obtained. Learning whether such an approach would work among couples in high HIV prevalence settings is vital for determining the full potential of self-testing as an HIV prevention.

Our intervention will consist of providing pregnant and post-partum women with multiple HIV self-tests so that they can then provide the tests to people who currently have lower uptake of HTC – their male partners. In this way, our intervention seeks to increase uptake of HIV testing among men.

# JUSTIFICATION

The proposed intervention helps overcome many of the barriers to uptake of facility-based HTC among men such as reluctance to visit healthcare facilities, stigma, and costs such as transportation and lost wages. The intervention leverages the advantages of self-tests – greater privacy and ease of use – while also using the female partners of men to advocate for HIV testing rather than other ways to promote testing among men that may be less effective. In the proposed intervention, women will be given two HIV self-tests and encouraged to either provide one self-test to their male partner for own use (partner testing) or to use the two tests together with their partners (couples testing). Since self-tests have advantages over facility-based HTC, the pilot intervention we will implement has the potential to achieve higher uptake of HIV testing among male partners than the current standard of care that encourages pregnant and post-partum women to refer their partners for HTC at fixed sites. The impact evaluation of the pilot intervention will use a randomized trial design to assess the effect of the intervention relative to the standard of care. Outcomes studied will include uptake of HIV testing among male partners, uptake of couples testing, reported adverse events, and linkage to care.

Our rationale for providing multiple self-tests to pregnant and postpartum women attending antenatal and postpartum clinics also lies in the special importance of partner or couples testing taking place in this population.Women have ongoing risk of acquiring HIV in pregnancy and postpartum [35]. Due to elevated risks of HIV transmission (female-to-male) and acquisition (male-to-female) during pregnancy – as well as the greater risk of maternal-to-child transmission if women have higher viral loads as a result of becoming newly infected during pregnancy or postpartum – it is particularly important that partners of pregnant women receive HTC. In fact, partner and couples HTC has been strongly endorsed by the WHO and fairly widely promoted in antenatal care settings [11]. The key challenge however, has been in promoting widespread *uptake* of either partner or couples testing in ANC settings. In most countries including Kenya only a small proportion of male partners attend ANC or postpartum clinics (PPC) with their partners and get tested with them [12, 36].

Novel data are also needed to determine whether the provision of multiple self-tests to women for the purpose of promoting partner or couples testing can be done without increasing the risk of intimate partner violence (IPV).One concern with the promotion of couples HTC in ANC settings has been IPV if one or both partners test HIV-positive. Results to date have been mixed; one randomized controlled trial (RCT) showed no effect of couples HTC on IPV whereas two observational studies found that women who received couples HTC reported more physical violence (3.3%) than women who receiving individual HTC (1.1%)[37-39]. It is important to establish whether IPV is any more likely to occur when women provide their partners with a self-test for individual use or when they initiate couples testing using self-tests. By virtue of being the first attempt to promote partner or couples testing using self-tests, our intervention and accompanying evaluation will provide much-needed evidence to determine whether such testing should be a paradigm that is adopted more widely.

Our intervention builds upon existing work that we have done to explore the acceptability and feasibility of an approach to provide self-tests to index persons for the purpose of promoting more widespread HIV testing in their sexual networks.With support from the Bill and Melinda Gates Foundation’s Grand Challenges Explorations program, our team has begun implementing a pilot intervention to explore the acceptability and willingness among women in Kisumu to use self-tests with their sexual partners. The early findings from our research will be essential for developing counseling protocols that accompany the intervention and developing strategies that facilitate the ability of women to bring up the issue of self-test usage with their partners.

# STUDY OBJECTIVES AND HYPOTHESES

This study will use a randomized controlled trial design withindividual-level random assignment to test whether offering multiple self-tests to women can increase the likelihood that either partner or couples testing takes pace. Our hypothesis is that the intervention group will have higher uptake of HIV testing by women’s partners within 3 months of enrollment in the study than the control group.

### Primary Outcome Measures

The primary outcome for the study will be uptake of HIV testing by women’s partnerswithin 3 months of enrollment in the study. For each participant, we will record whether the participant’s partner underwent HIV testing – in the case of the intervention group, this will be a report by female study participants that their partner used a self-test; in the control group, this will be uptake of HTC at the VCT by the partner. We will test for differences in the primary outcome variable between the intervention and control group.

### Secondary Outcome Measures

The secondary outcomes for the study will further describe uptake of HTC in the intervention and control groups. In particular, we will compare the following outcomes between the two study groups:

Discussions about and uptake of HIV testing:

- Proportion of participants who report that they discussed HIV testing with their partner;
- Proportion of participants whose partners undergo HIV testing individually (partner testing);
- Proportion of participants who undergo couples HIV testing with their partners;

In accordance with WHO guidelines, we define partner testing as a scenario in which one partner (for example, women who participate in our study) has already tested and the other partner (the primary sexual partner of the women) is tested separately without mutual disclosure of HIV status necessarily occurring. On the other hand, couples testing is defined as a scenario in which both partners are counselled, tested, and provided with their results together so that mutual disclosure of HIV status occurs.

Actions taken after HIV testing:

- Sexual behavior and decision making (such as condom use) after HIV-positive and HIV-negative test results for partners;
- Adverse reaction to a positive or discordant result

Since confirmatory testing is recommended following the use of HIV self-tests, in the intervention groupwe will also measure *use of confirmatory testing*by individuals who were reported to have used the self-tests. Participants will be given referral vouchers for confirmatory testing at several VCT clinics in Kisumu so that this can be recorded. We will also obtain self-reported information from the study participants about whether each person who used the self-test sought confirmatory testing.

We will also describe several aspects of self-test usage (where it takes place, how often women suggest the self-test usage to their partners, what reactions male partners have to self-test, etc.).During the follow-up interview we will ask to see the used self-testing package if study participants report that they or their male partner used the self-test.

We will also report outcomes related to social harms, such as experiences of IPVand increase in unprotected sex, if they occur.

### QualitativeSub-Study

In addition, a qualitative sub-study focused on women in the intervention group will provide additional details on the usage of self-tests for promoting partner or couples HIV testing. We will conduct in-depth interviews with participants and explore in-depth the process of making a decision to suggest use of the self-test with a sexual partner; the reactions of sexual partners; the experience of using the test with a sexual partner; and decisions made after using the self-test, including sexual behavior decisions, experiences of violence or coercion, uptake of confirmatory testing, and linkage to care.

# STUDY DESIGN

## Overview of study design

To determine the impact of the intervention on partner or couples HIV testing, the project will use a randomized controlled trial design.

We will recruit about 600 adult womenfrom antenatal and postpartum careclinics. Participants will be randomized to one of two groups: intervention or control.

Following a baseline interview, participants in the intervention groupwill be shownhow to correctly use theself-tests and given two HIV self-tests. Subsequently, we will contact participants periodically over a 3 month period to see if they have used the test(s) with their sexual partners and conduct a follow-up interview.

Following the baseline interview, participants in the control group will be given referral vouchers for themselves and their partners to obtain HIV testing at VCT centers. We will contact the participants at the end of 3 months to see if they and/or their partner(s) have sought HIV testing.

We will also conduct in-depth qualitative interviews with a subset of study participants in the intervention group, including participants who have used HIV self-tests together with their partner, those who have used the test [partner testing] and those who have not used HIV self-tests.

## Study site

The study will take place in Kisumu, Kenya. Adult HIV prevalence in the Nyanza region where Kisumu is located is the highest in Kenya at 15.1%.As the primary urban and commercial area in Nyanza region, Kisumu has even higher prevalence than many other parts of Nyanza [40]. Identifying new and effective strategies for HIV prevention is therefore urgently needed in this region.

The intervention will be implemented at 3 health centres in Kisumu: the Lumumba Health Centre (LHC), Kisumu East District Hospital (KEDH), and Rabuor Health Centre. LHC is owned and operated by the City Council of Kisumu While both KEDH and Rabuor are government facilities managed by the Ministry of Health (MoH).

LHC facility is one of the largest in the whole of Kisumu County, seeing an average of 400 women per month for ANC and 60 women per month for PPC. Besides the robust ante- and post-natal services, LHC also provides antiretroviral therapy, family planning, HIV counseling and testing, and child immunization. LHC hosted sites for numerous prominent clinical trials, including the University of Illinois-University of Nairobi Kisumu male circumcision trial and the University of Washington-Kenya Medical Research Institute HSV-2 and Partners PrEP trials.

KEDH is a Ministry of health facility in Kisumu within the City of Kisumu, serving mostly residents of the City and its environs. The facility offers both out and in-patient curative services. Other services offeredinclude: Antiretroviral Therapy, Family Planning, HIV Testing and Counseling and, Immunization. About 800 women come to the facility for ANC and 80 women for PPC every month. In the month of October 2014, a total of 998 women received family planning (FP) services from KEDH out of which 382 were seeking FP services for the first time.

Rabuor Health Center is a Ministry of health facility in Kisumu East, sub-county, Kadibo Location. It is about 12 Kilometers from Kisumu town and services offered at the facility include: Antiretroviral Therapy, Family Planning, HIV Counseling and Testing and Immunization, among others. Its catchment area includes, Masogo, Kochieng, Okana, Nyamware South and Nyamware North Locations. Every month the facility serves about 250 and 60 women at the ANC and PPC respectively.

## Study population

We will seek to enroll 600adult women who are attending the health centres’ antenatal or post-partum care clinics. One half of the women in each site will receive the intervention described in this section and the other half will receive the standard of care.

## Inclusioncriteria

To be eligible to receive the intervention, women must meet the following inclusion criteria:

1. 18-39 years of age
2. Primarily resides in or near Kisumu
3. Reports having a current, primary partner (someone that the woman has been in a relationship with for at least 6 months and is identified as her primary partner)
4. Have undergone the opt-out HTC at the ANC or PPC clinic
5. Intends to continue living in or around Kisumu for the next 3 months (follow-up period).

## Exclusion criteria

Participants who meet the following criteria will notbe eligible for enrollment:

1. Male
2. Age <18years or >39years
3. Believes that her partner will hurt her if she gives him an HIV self-test and encourages him to use it
4. Reports having an HIV+ partner
5. Intends to relocate from Kisumu area within the next 3 months

## Sampling

Research Assistants (RAs) will invite eligible individuals to participate in the study.Recruitment of participants will take place at the Lumumba Health Centre in Kisumu, Kisumu East District Hospital and Rabuor Health center. Staff at the ANC and PPC clinics will inform patients as they are being discharged home that there is a study they may qualify for; if they express interest in learning more, they will be given a referral coupon to take to the study staff based at the facility. For ANC and PPC patients, the referral coupons will be identical and will have a unique number rather than the women’s names or any other identifying information. Before issuing the coupon to women, the ANC and PPC staff will write down a discreet code for whether the woman is HIV-positive or HIV-negative (OP for HIV-positive and ON for HIV-negative). Only the study and clinic staff will know what the codes denote. We believe the coding system will be discreet enough to not disclose the HIV status to unauthorized person(s). This information will be necessary for assessing participation rates in the study by HIV-positive and HIV-negative women, analyzing use of self-testing by HIV status, and for sampling HIV-positive and HIV-negative participants in qualitative interviews. Once the RAs collect the referral coupons from the women, the number on the coupon and the HIV status code on the coupon will be recorded on secure tablets. No identifying information will be collected for women who decline to participate in the study.

Women at all stages of pregnancy and post-partum care will be referred to study RAs. The study RAs will then explain the study to women, screen them for eligibility for the study, and ask those who are eligible if they are willing to participate in the study. RAs will obtain informed consent and enroll the woman into the study. We will conduct recruitment in the study facilities in a manner that ensures the number of participants in each facility is proportional to the number of women who seek care at each facility.

During the qualitative phase of the study, we will purposively sample a sub-set of about 45 study participants. We will seek to include 10-15 women who tested together with their partners (couples testing), 10-15 women who gave the test to their partner to use (partner testing), and 10-15 women who never used the test or gave it to their partner to use.We will sample from among all study sites and both ANC and PPC clients.

## Procedures

The intervention will consist of providing multiple HIV self-tests to women attending an ANC or post-partum clinic (PPC) in the city of Kisumu and its environs, providing information to them on how to use the self-tests and where to go for confirmatory testing and care if necessary, and then encouraging them totalk to their partner about self-testing and ask their partner to use the self-test (either independently or together).

The effect of this strategy on uptake of HIV testing among male partners will be compared with the current standard of care in which women are encouraged to refer their partner for HTC. After a 3 month period, we will obtain information from the women on the receptivity of their sexual partner to self-testing, whether they were able to use self-tests with their partner jointly or independently, and the incidence of any adverse events; participants will be encouraged to report any adverse event as soon as it occurs. Uptake of partnerHIV testing will be compared between self-testing and provider-based testing. We will also record whether the women’s partners seek confirmatory testing and link to care if an HIV-positive test result is reported to be obtained.

**Baseline data collection**

After determining that women meet the above eligibility criteria, trained RAs will obtain informed consent and then administer a baseline questionnaire,educate participants on how to use HIV self-tests (as discussed in Section I), and then give each participanttwo HIV self-tests. This will be conducted in a private location within each health facility.

In the baseline interview, we will collect data on demographic characteristics, employment, income, sexual behavior history, bargaining power in the couple, self-efficacy measures, HIV/STI testing history, partnership history, and IPV history. The purpose of the interview will be to obtain basic demographic and socio-economic information, sexual behavioral history, and history of HIV testing. Trained RAs will administer questionnaires using encrypted, password-protected tablet computers that contain forms developed using Open Data Kit (ODK), an open-access data collection platform that we have used in other studies.

**Randomization**

Following completion of the interview, each participant will be randomly assignedto one of the two study arms.We will conduct 1:1randomization in blocks of 20 study participants so that an equal number of participants will be randomized to the control group and intervention group. The group to which each participant is assigned will be recorded by the RAs and linked to the participant’s study ID number.

**Intervention group**

Participants in the intervention group will be given clear, accessible instructions on how to use the tests (this will be communicated to them and provided on a written document). Participants will be instructed to call a phone hotline that we will establish in the event of any adverse events that they or their sexual partners experience. They willalso be given a list of places that partners can access for confirmatory testing and advisedto encourage their partner to go for confirmatory testing.

They will be also receive information about HIV self-testing including reasons for suggesting usage of the self-test to their partner and strategies for introducing self-tests to their partners; however, it will also be emphasized that women are under no obligation to suggest to their partner or convince him to use the self-test. Because use of self-tests at home will mean that a trained counselor will not be present when women or their partners take the test, we will develop messages that can be communicated to women easily so that they and their partners can use the self-tests correctly and safely on their own. The messages and counseling that we will provide will adhere to Kenya’s National Guidelines for HIV Testing and Counseling [16].

Two self-test kits will then be distributed to women, each containing the following:

- One standard OraQuick ADVANCE I/II test kit with developer fluid vial and stand;
- Written pre-test information including test instructions in written and pictorial form, post-test counselling information and details about how and where to access HIV confirmatory testing in or near Kisumu;
- Aphone number that participants can call to obtain additional information about taking the test, interpreting results, obtaining referral information,post-test counseling or obtain help in case of IPV.
- Two voucherswith unique identifying numbers which can be redeemed at confirmatory testing for a small token.

All the information above will be communicated orally to women initially and also provided in written form ofan information sheet, in English, Kiswahili and Luo.

**Control Group**

The control group will get standard of care ante/post-natal care as per the MoH guidelines whereby pregnantwomen are encouraged to learn their HIV infection status, as well as that of their sexual partners.For postnatal women, HIV counseling and testing is usually offered to all women with unknown HIV status. In the study, all women in the control group will be given referral coupons with unique identifying numbers which they (and their partners) can redeem at a VCT center for a small token. Participants will be encouraged to go to one of the study facilities for HIV testing.

**Follow-up interviews**

Follow-up interviews with participants in the control and intervention groups will be conducted by trained counselors 3 months after enrollment in order to learn about decision-making regarding partner or couples testing. In the control group, we will obtain information from participants on whether they asked their partner to accompany them to the ANC and use the referral voucher for voluntary HTC. In the intervention group, we will obtain information from participants on whether they suggested usage of the self-test to their partner, whether their partner used the self-test, and whether the couple used self-tests together (with mutual disclosure of HIV status) or if instead the partner was believed to have used the self-test alone. In cases where self-tests were reported to have been used, trained HTC counselors will also ask to see the self-tests and obtain visual confirmation of usage. We will also obtain self-reported information on whether partners who used self-tests sought confirmatory testing (those who did not but wish to have their results confirmed during the visit will be tested by the counselor) and whether partners who obtained an HIV-positive test result sought care at an HIV clinic.

In both groups, we will ask participants whether their partners have accompanied them for ANC or PPC visits in the past 3 months. We will also ask participants if their partners have sought care at an HIV clinic in case an HIV-positive test result was obtained. Finally, in both groups, we will inquire about adverse eventssuch as reports of verbal abuse or physical violenceduring the follow-up interview; those who report events will be referred for support. The study will provide a 24-hour hotline that participants can call with questions or to report an adverse event. The hotline will be staffed by trained study personnel who will be available to counsel and provide referrals to participants. If any adverse events are reported, they will immediately be reported to the Ethical Review Boards and action will be taken where possible.

**Recording uptake of voluntary HTC (control group)**

At the time of enrollment participants in the control group will be provided with referral vouchers (with a unique participant ID code) for HTC (at LHC or another facility of their choice within and around Kisumu) that they and their partners can use for either partner testing (partner tests alone) or couples testing. In the 3 months after enrollment, we will be able to clearly determine whether participants’ partners came for HTC either individually or as a couple based on whether the referral vouchers were presented at HTC sites.

Research assistants at LHC, KEDH and Rabuor Health Centrewill collect and record the referral voucher numbers (with a unique participant ID code) presented at the HTC sites when an individual comes for HIV testing. They will record whether participants’ partners presented these vouchers in the 3 months after enrollment in order to obtain information on utilization of testing by both intervention and control groups. This strategy has been successfully implemented in previous studies that our team has conducted in the Nyanza region of Kenya, including impact evaluations supported by the Gates Foundation and 3ie in which we record uptake based on presentation of referral vouchers.

**Utilization of confirmatory testing (intervention group)**

In the intervention group, participants will be provided with referral vouchers (with a unique participant ID code) that they and their partners can use for confirmatory testing (at any of the three study facilities or another facility of their choice within and around Kisumu). We will record whether participants’ partners presented these vouchers in the 3 months after enrollment in order to obtain information on utilization of confirmatory testing.

**Qualitative interviews**

We will conduct a qualitative sub-study in which we will explore in-depth the process of making a decision to suggest use of the self-test with a sexual partner; the reactions of sexual partners; the experience of using the test with a sexual partner; and decisions made after using the self-test, including sexual behavior decisions, any violence, uptake of confirmatory testing, and linkage to care.

We will conduct in-depth interviews with 45 participants from the intervention group. We will purposively sample to include 15 participants who report using self-tests as a couple, 15 who report their partner using self-tests independently, and 15 who chose not to use self-tests. We will also seek to include women who attended antenatal care and postpartum care, as well as both HIV-positive and –negative women to achieve maximum variation among qualitative participants.After the 3-month follow-up interview, the study coordinator will identify participants for the qualitative interviews in the above three categories. The study coordinator will then contact the selected participant to set up interviews. Interviews should be scheduled to take place within 1 – 2 weeks of scheduling. The interview will take approximately one hour and will be conducted at the participant’s home, the IRDO office, or another location convenient to both the participant and RA.

The qualitative data will be used to better understand women’s ability to encourage partner testing with the self-tests and gain insights on reasons why it was and was not possible for them to suggest self-test usage to their partners. The qualitative data will also enable to use to gain insights from study participants on the way their male partners reacted to the use of self-testing and explore social harms. All these elements will be important for interpreting results from the quantitative study.

## Study withdrawal for participants

Participants will be free to withdraw from the study at any time. Reasons for study withdrawal may be ascertained.

## Adverse events

Because participants will above all be making voluntary decisions to introduce self-testing with their sexual partners, we do not expect to have significant adverse events related to the usage of the self-test with partners.However, we are concerned about the safety of study participants and have prepared for the unlikely event of violence as a result of introducing the self-test to a sexual partner. During initial enrollment and counseling, we will include referral information regarding services for those experiencing IPVand we will also advise participants to use their best judgment of whether they feel comfortable introducing self-testing with a sexual partner. We are excluding from the study women who express concern that they would experience IPV as a result of suggesting HIV self-test use.Because it is possible that the sexual partners of a participant will react adversely to an HIV-positive test result, we will provide detailed oral and written information to participants on locations where IPV services, confirmatory testing, and HIV care and treatment are available. We will also set up a phone hotline for the study which will be available 24 hours per day. This hotline will be operated by the study staff, who will be available to answer questions related to the study, the use of self-test, as well as locations where participants or their sexual partners can be referred to for IPVservices, for confirmatory testing, and for HIV care and treatment. The telephone hotline will be established for use by the participants free of charge to them. Participants will be asked to “flash” the hotline number, i.e. dial the hotline and disconnect after a single ring thereby alerting the holder of the hotline that the participants is asking to be called back. Alternatively, they will be informed to send “Please call me” and the study coordinator will call them back.

Every effort will be made to avoid violence. During the training session, participants will be instructed about assessing client behavior and if a participant is concerned that a client will be violent, she will be counseled not to introduce self-testing with that partner. However, violence from partners may occur and there are measures in place to deal with such cases. For example in cases of rape or non-consensual sex, post-exposure prophylaxis can be administered and trauma counseling done at Impact-RDO Drop In Center (DICE) in Kisumu (TumainiNaselica DICE). Participants who experience violence will be referred to Violence Protection Centre at JOOTRH (JaramogiOgingaOdinga Teaching and Referral Hospital, Kisumu) for medical care (if necessary) and psychosocial services. Other options include reporting to the police for possible legal action. All these options will depend on the complainant. All of this information will be shared with participants during the counseling session and also available via the hotline number.

# ETHICAL CONSIDERATIONS

## Informed Consent

Before studyenrollment, womenwill be introduced to the study and its aims, as outlined in the informed consent document. The consent forms will describe the study procedure in the language (English, Swahili or Dholuo) preferred by the participant. The subject will be asked to read and review the document. Interviewers will be careful to impress upon subjects that they may choose to decline participation or withdraw from the study at any time. Those who agree to participate in the study will be asked to sign and date the informed consent form prior to enrollment and random assignment. If a subject cannot read, the consent form will be read to them and oral consent will be obtained,with a witness providing his or her signature. A copy of the informed consent document will be given to the subjects for their records.

## Ethical Approval

All study participants will be fully informed of the study procedures as described above. Ethical approval for this study will be sought from the Kenya Medical Research Institute’s (KEMRI) Ethical Review Committee and the Institutional Review Board at UNC-CH.

## Protecting privacy and confidentiality

All study staff will undergo ethics trainingand sign a confidentiality agreement (Appendix7: Confidentiality statement). All education, counseling and testing will be done as stipulated in Kenya’sNational Guidelines for HIV testing and counseling. All participants will be identified using a unique identification code that will be assigned to them at the first visit.

All electronic datacollection will take place on encrypted, password-protected tablet computers. Data will be sent to encrypted servers and will only be accessible to authorized study personnel. Consent forms and other paper materials will be stored in locked cabinets with access restricted to authorized study personnel.

## Potential risks of proposed research to study participants

We anticipate minimal risks to study participants. Although there is a potential for exposing a participant’s personal information to others during the interview, every consideration will be taken to ensure the interview is conducted in a private place. Study procedures will be thoroughly explained to participants and they will be consented only if they fully understand study procedures and find them acceptable. Participants are allowed to exit from the study at any time.

We do not anticipate any adverse events following the self-testing, outside normal reactions similar to those occurring among persons testing for HIV the first time.These may include negative reactions to an HIV diagnosis or incorrect test results. Participants will be advised to visit a voluntary counseling and testing (VCT)clinic for a confirmatory test as the self-test is a primarily a screening test. The participants will be well trained on self-testing and counseled before being given the test kits. They will also be given a phone number they can call at any time.The information given will instruct the participants about the potential benefits to using the test prior to sex with a partner (for example, knowing partner’s status before sex can lead to better risk reduction). They will also practice and become comfortable using the kits during this session. All of this will take place at the time of enrollment.

There is a risk that sexual partners will react adversely to the participants’suggestions of self-test usage or to the test result itself. To minimize this risk, we will provide detailed information on the need for confirmatory testing and the availability of HIV care and treatment for HIV-infected persons. We will not enroll women who self-report concern that their partner may hurt them if they suggest using the HIV self-test and in addition we will refer men and women who experience violence forto IPV services.

## Potential benefitsof proposed research to study participants and others

Study participants will havethe opportunity to receiveHIV self-tests to use themselves and to use with their sexual partners. The benefits to participants include increased knowledge about their own HIV status and potentially their partners’ HIV status, which in turn may serve to reduce their risk of HIV infection.

The knowledge gained from this study will also help inform policy makers and HIV prevention programs about the potential role that HIV self-tests can play in increasing awareness of HIV status and reducing the number of new HIV infections.

1. **DATA MANAGEMENT**

## Data storage

All study computers and records will be stored in a secure room with access limited only to authorized staff. All computers and databases will be encrypted and password-protected with limited access. Daily backup of the data will be done by a designated study staff onto a secure server at UNC-CH.

Data collection forms will be stored at IRDO HQ in Kisumu, which will be the operating headquarters of the study. No participants will have any identifier on the data forms; names and signatures will only be on the consent forms, which will be kept under lock and key by the Study Coordinator and after signing, will be accessible only to the PIs, Coordinator and IRDO’s Research Officer. All electronic data collection will take place on encrypted, password-protected tablet computers. Data will be sent to and stored on encrypted, password-protected servers at UNC and will only be accessible to authorized study personnel. At the end of the study, data will be kept for up to 5 years for electronic version and up to 2 years for paper forms, including consent forms. The site PI (KA) will take responsibility of all study procedures, including long-term storage and disposition of data.

## Data management

All statistical analyses will be performed using Stata.

Among the women screened for eligibility, we will first tabulate the reasons for ineligibility.

**Baseline data**

We will use the baseline data to summarize various characteristics of study participants, including age, marital status, education, sexual behavior, and recent HIV testing history. We will report various statistics of the quantitative variables (mean, median, variance, range, IQR, etc.) as well as frequencies for the categorical variables.

**Follow-up data**

We will use the follow-up data to determine the following statistics for each group of participants:

1. Proportion of partners who report that their partner used an HIV self-test
2. Proportion of participants who report that they discussed HIV testing with their partner
3. Proportion of participants whose partners undergo HIV testing individually (partner testing)
4. Proportion of participants who undergo couples HIV testing with their partners
5. Proportion of participants who report mutual disclosure of HIV status with their partner
6. Proportion of participants who reported making sexual behavior decisions (sexual intercourse, condom use) on the basis of a sexual partner’s decision to use or not use the self-test and on the basis of a sexual partner’s test result
7. Proportion of participants who reported that their partner sought confirmatory testing after using the self-test
8. Reports of social harms, such as IPV or coercion as a result of discussing or using HIV self-test

**Data Security**

We will develop Standard Operating Procedures (SOPs) for data security and confidentiality procedures at collection, transfer, entry and storage levels, and make these readily accessible to all staff members who have access to confidential study data. All paper data will be kept in a lockedcabinet. Electronic data will be kept on encrypted and password protected computers. Access to confidential data will be limited to authorized persons, and released data will contain no identifiable information.

**Qualitative data**

Qualitative interviews will be audio-recorded, transcribed, and translated. Data will be coded and analyzed utilizing Nvivo software. Analysis will be iterative, beginning during data collection so that the interview guide and purposive sampling can be refined as needed. A team-coding approach will be used and analysis will be refined through discussion with the team members and consensus building. Thematic analysis will then conducted to answer the research questions. The qualitative data will be used in conjunction with the quantitative data to describe the use of self-testing kits with partners.

# STATISTICAL CONSIDERATIONS

## Statistical analysis

Our primary analysis will use logistic regression to report unadjusted and adjusted odds ratios for the effect of the intervention on uptake of HIV testing by the primary partners of study participants. The primary outcome variable (defined in detail above) will be a binary variable indicating whether or not the partner of each participant was reported to have tested for HIV (either with a self-test in the case of the intervention group or at a VCT in the case of the control group). Logistic regression models will be estimated in STATA with and without controls for age, education, indicator of whether the participant is seeking antenatal or postpartum care, and partner characteristics (age, education, employment status). All other statistical tests that compare the intervention and control groups will be 2-sided and significance will be set at P < 0.05.

In secondary analyses, we will also estimate logistic regression models in which the effect of the intervention on secondary outcomes defined above is studied. For example, we will examine whether HIV testing was more likely to be suggested to primary partners in the intervention or control groups. Similarly, we will assess the effect of the intervention on other secondary outcomes including mutual disclosure of HIV status and incidence of adverse events.

Finally, to assess the potential for using self-testing to promote HIV testing among male partners of HIV-infected and HIV-uninfected women, we will also carry out analyses in which we compare whether the uptake of HIV testing by participants’ partners and mutual disclosure differs between HIV-infected and HIV-uninfected women.

We will conduct sensitivity analysis using inverse probability weighting to correct for selective uptake of the intervention. This robustness test predicts the probability of intervention uptake using the baseline variables and reweights the estimates to make them more representative.

## Sample size justification

Power calculations were performed using the “sampsi” and “sampclus” commands in Stata version 13.1 and included adjustments for the design effect associated with enrolling participants in multiple facilities. These calculations suggest that with 20% of male partners in the control group seeking either partner or couples HTC at the Lumumba Health Centre within 3 months, a sample size of 300 women per group (600 women in total) will provide adequate statistical power to conduct pair-wise comparisons. A sample size of 300 women in the intervention group and 300 women in the control group will provide 80% power to detect a difference in use of partner or couples HTC as small as 10%. Given that partner or couples HTC is expected to be 20% in the control group, we believe that a 10% increase (to 30%) is sizable and meaningful from a policy standpoint.

We believe partner testing of 20% in the control group is not unreasonable in light of other studies (conducted several years ago when acceptance of HIV testing among male partners was lower) showing that 14-16% of ANC attendees who were invited to return to the clinic with their partners successfully returned with them and that nearly all those partners accepted HIV testing. We also hypothesize that the difference in HIV testing in the intervention group will be sizable in light of the added convenience of HIV self-tests and the high level of acceptability of self-tests in Kenya.

# LIMITATIONS

This study has several limitations that warrant discussion. We recognize the limitations of relying on self-reported HIV testing as an outcome variable. This is an inherent issue in all studies to date that have measured the use of self-tests, since the self-tests are typically used in private. We will take several steps to overcome the limitations of having self-reported outcomes in the intervention group and an objective outcome in the control group. First, in the intervention group, during the follow-up interview we will ask to see the used self-testing package in cases where study participants report that they or their male partner used the self-test (this has previously been done in other studies in Malawi). While the verification of used self-testing packages is not necessarily a flawless measure of actual usage of self-testing, it will provide some indication of how much higher the self-reported measure is relative to the verified measure. Second, we will also explore ways to verify whether the participants’ partner sought confirmatory testing after using the self-test: If the participant states that their partner went for a confirmatory test, we will check whether the referral voucher given to them were recorded at the VCT that they visited.

In comparing the intervention and control groups, we will also examine a self-reported outcome that will be similar in both study arms so as the reduce bias in the estimated effect of the intervention: in particular, we will ask study participants in both groups to self-report whether or not their partner tested for HIV through any modality after enrollment in the study (either in clinic or with self-testing). By virtue of being self-reported and comparable in both study arms, this will enable us to determine if the intervention resulted in higher reported HIV testing for ther partners of study participants. We believe that when we triangulate all this information, we will be able to make a more accurate determination of whether the intervention is effective in promoting HIV testing.

# DISSEMINATION AND EXPECTED APPLICATION OF THE RESULTS

The results will be shared with the LHC staff and other stakeholders in the community; Tumaini DICE staff, Peer Educators, study participants and other FSWs; NASCOP and other stakeholders in relevant forums, such as the MoH meeting in Kisumu County. The results will also be presented at local and international meetings and conferences where other researchers and policymakers are present, particularly those working on HIV prevention and treatment. In addition, we will prepare manuscripts containing our quantitative and qualitative results and submit them to peer-reviewed journals for publication and wider dissemination.

This study will provide important data on the feasibility and impact of an intervention that relies on HIV self-tests to increase HIV testing among men and among couples. The intervention is novel because it uses pregnant and postpartum women (who have high acceptance of HIV testing) to promote HIV testing among male partners. Improving coverage of HIV testing is a critical goal in HIV prevention and treatment programs. Adult men are less likely to seek HTC than women, and finding novel ways to close the male testing gap is a major priority. With the recent introduction of HIV self-testing in Kenya and the high coverage of HIV testing among women through antenatal clinics, this study will examine a timely and novel application of self-testing to increase testing among men. Increasing HIV testing among Kenyan men and among couples has the potential to reduce new infections, enable discordant couples to access interventions that reduce HIV transmission, reduce the incidence of mother-to-child transmission, and improve treatment outcomes for HIV-positive individuals through early testing and treatment.

The rigorous impact evaluation design we have proposed will mean that our findings will be of relevance to Kenya’s MOH as it seeks to achieve increased HIV testing among men. Our study will provide policy makers with data about whether self-tests can be used to increase male testing for HIV and reduce HIV incidence through knowing ones’ status, timely treatment, and sexual risk reduction after testing. We have also incorporated qualitative methods into this study in order to better understand how and why the intervention does or does not work. If successful, this intervention would provide a more effective way of reaching men. We believe there is high potential for this intervention to be implemented in multiple settings. HIV self-testing with sexual partners, if effective, may have implications for other populations such as young women and adolescent girls at risk for HIV, commercial sex workers, and MSM. Furthermore, if the intervention is found to be ineffective, this impact evaluation will be useful for guiding public health decisions about where and how self-testing should be promoted.

# STUDY TIMELINE

The period planned for the project is13 months as shown in the tablebelow:

**Table 1: Study Timeline**

1. **BUDGET**

**Table 2: University of North Carolina Budget**

| *CATEGORY* | *AMOUNT (USD)* |
| --- | --- |
| Personnel Costs | 122,192 |
| Travel Costs | 12,750 |
| Other direct costs | 15,978 |
| Indirect cost | 17,375 |
| **TOTAL** | **168,295** |

**Table 3: Impact Research and Development Organization Budget**

| *CATEGORY* | *AMOUNT (USD)* |
| --- | --- |
| Personnel Costs | 43,284 |
| Travel Costs | 1,120 |
| Materials & Supplies | 65,249 |
| Other direct costs | 4,478 |
| Indirect cost | 17,120 |
| **TOTAL** | **131,250** |

1. **APPENDICES**

Appendix 1:Screening Questionnaire

Appendix 2: Written Informed Consent

Appendix 3: Baseline Questionnaire

Appendix 4:Oraquick instructions

Appendix 5: Follow-up Questionnaire

Appendix 6: Written Informed Consentfor In-depth Interview

Appendix 7: In-depth Interview Guide

Appendix 8: Confidentiality Statement

# ROLES AND RESPONSIBILITIES

The study is being conducted by Drs. Harsha Thirumurthy and KawangoAgot, with support from the Bill and Melinda Gates Foundation.

| **Title** | **Role** | **Responsibilities** |
| --- | --- | --- |
| PIs | Dr. Harsha Thirumurthy  Dr. KawangoAgot | Dr. Thirumurthy and Dr. Kawango will lead the study and share responsibility for implementation of the study according to protocol.  Dr. Kawango will work with the Study Coordinator and Dr. Omanga to hire, train and supervise RAs who will be enrolling and interviewing participants; prepare weekly and other scheduled and un-scheduled progress reports; track clients.  Dr. Thirumurthy will receive weekly field reports. He will also take responsibility for developing data collection tools, monitoring data quality, analyzing the data and writing up the results. as well as from the part-time Project Manager based at UNC (Kate Murray) |
| Co-Investigators | Dr. Eunice Omanga  Ms. Immaculate Akello  Dr. Suzanne Maman  Dr. Sue Mavedzenge  Ms. Kate Murray  Dr. Arianna Zanolini | Dr. Omanga and Ms. Akello will hire, train and supervise field staff who will be enrolling and interviewing study participants; prepare weekly and other scheduled and un-scheduled progress reports. Ms. Akello will be responsible for study coordination on a day to day basis.  Dr. Maman and Dr. Mavedzenge will advise the study team on qualitative research methods and issues related to HIV testing.  Ms. Murray will assist the development of data collection tools, data monitoring, and data analysis. She will review the database and provide monitoring support to Dr. Omanga and Ms. Akello.  Dr. Zanolini has experience implementing self-testing studies in Zambia and will contribute to development of data collection procedures and data analysis. |

REFERENCES

1. Baggaley, R., et al., *From caution to urgency: the evolution of HIV testing and counselling in Africa.* Bull World Health Organ, 2012. **90**(9): p. 652-658B.

2. Statistics, K.N.B.o., *Kenya Demographic and Health Survey 2008-09*, K.N.B.o. Statistics, Editor. 2010: Nairobi.

3. Donnell, D., et al., *Heterosexual HIV-1 transmission after initiation of antiretroviral therapy: a prospective cohort analysis.* Lancet, 2010. **375**(9731): p. 2092-8.

4. Cohen, M.S., et al., *Prevention of HIV-1 infection with early antiretroviral therapy.* N Engl J Med, 2011. **365**(6): p. 493-505.

5. Baeten, J.M., et al., *Antiretroviral prophylaxis for HIV prevention in heterosexual men and women.* N Engl J Med, 2012. **367**(5): p. 399-410.

6. Jaen, A., et al., *Determinants of HIV progression and assessment of the optimal time to initiate highly active antiretroviral therapy: PISCIS Cohort (Spain).* Journal of acquired immune deficiency syndromes, 2008. **47**(2): p. 212-20.

7. Kitahata, M.M., et al., *Effect of early versus deferred antiretroviral therapy for HIV on survival.* N Engl J Med, 2009. **360**(18): p. 1815-26.

8. Rosen, S., M.P. Fox, and C.J. Gill, *Patient retention in antiretroviral therapy programs in sub-Saharan Africa: a systematic review.* PLoS medicine, 2007. **4**(10): p. e298.

9. Sterne, J.A., et al., *Timing of initiation of antiretroviral therapy in AIDS-free HIV-1-infected patients: a collaborative analysis of 18 HIV cohort studies.* Lancet, 2009. **373**(9672): p. 1352-63.

10. Ng'ang'a, A., et al., *The status of HIV testing and counseling in Kenya: results from a nationally representative population-based survey.* Journal of acquired immune deficiency syndromes, 2014. **66 Suppl 1**: p. S27-36.

11. World Health Organization, *Guidance on couples HIV testing and counselling including antiretroviral therapy for treatment and prevention in serodiscordant couples: recommendations for a public health approach*. 2012, World Health Organization: Geneva.

12. Katz, D.A., et al., *Male perspectives on incorporating men into antenatal HIV counseling and testing.* PLoS ONE, 2009. **4**(11): p. e7602.

13. Gelmon, L., et al., *Kenya HIV Prevention Response and Modes of Transmission Analysis*. 2009, National AIDS Control Council: Nairobi.

14. Obermeyer, C.M. and M. Osborn, *The utilization of testing and counseling for HIV: a review of the social and behavioral evidence.* Am J Public Health, 2007. **97**(10): p. 1762-74.

15. Hutchinson, A.B., et al., *Understanding the patient's perspective on rapid and routine HIV testing in an inner-city urgent care center.* AIDS Educ Prev, 2004. **16**(2): p. 101-14.

16. National AIDS and STD Control Programme, *Guidelines for HIV Testing and Counselling in Kenya*, Ministry of Public Health and Sanitation Kenya, Editor. 2008, Nairobi: NASCOP.

17. Mukoma, W. *Exploring potentially effective methods for counselling and linkage to care in the context of HIV self-testing in Kenya*. in *3ie Matchmaking Meeting 28 April 2014*. 2014. Nairobi, Kenya.

18. Corbett EL, *Health worker access to HIV/TB prevention, treatment and care services in Africa: situational analysis and mapping of routine and current best practices*, in *Final 5 country report*. 2007, London School of Hygiene & Tropical Medicine; WHO/HIV department; WHO/TB department; Global Health Workforce Alliance.

19. Kalibala, S., W. Tun, and W. Muraah, *Feasibility and acceptability of HIV self testing among health care workers: results of a pilot programme in two hospitals in Kenya*, in *XVIII International AIDS Conference*, Abstract WEPDC205, Editor. 2010: Vienna, Austria 18-23 July.

20. National AIDS and STD Control Programme, *Preparedness for HIV/AIDS service delivery: The 2005 Kenya Health Workers Survey*, Ministry of Health Kenya, Editor. 2006, Nairobi: NASCOP.

21. Carballo-Dieguez, A., et al., *Will Gay and Bisexually Active Men at High Risk of Infection Use Over-the-Counter Rapid HIV Tests to Screen Sexual Partners?* J Sex Res, 2012.

22. Lee, V.J., et al., *User acceptability and feasibility of self-testing with HIV rapid tests.* Journal of Acquired Immune Deficiency Syndromes: JAIDS, 2007. **45**(4): p. 449-53.

23. Gaydos, C., et al., *Can we ever expect to have individuals perform their own HIV rapid tests?*, in *Infectious Diseases Society of America (IDSA); Abstract 180*. October 21-24, 2009: Philadelphia, PA.

24. Spielberg, F. and G. Foundation., *Acceptability of Self-Testing for STIs in India.* Abstract, 2010.

25. Choko, A.T., et al., *The Uptake and Accuracy of Oral Kits for HIV Self-Testing in High HIV Prevalence Setting: A Cross-Sectional Feasibility Study in Blantyre, Malawi.* PLoS medicine, 2011. **8**(10): p. e1001102.

26. MiraTes Europe BV, *HIV Home testing, the key to reaching high risk groups*. 2008.

27. Sharma, A., P.S. Sullivan, and C.M. Khosropour, *Willingness to take a free home HIV test and associated factors among internet-using men who have sex with men.* Journal of the International Association of Physicians in AIDS Care, 2011. **10**(6): p. 357-364.

28. Gaydos, C.A., et al., *Will patients "Opt In" to perform their own rapid HIV test in the emergency department? (Special Issue: HIV screening in the emergency department.).* Annals of Emergency Medicine, 2011. **58**(1): p. S74-S78.

29. Morgan, D., *FDA panel backs OraSure's In-Home HIV test*. May15, 2012; Accessed May 16, 2012; <http://www.reuters.com/article/2012/05/15/us-usa-aids-orasure-idUSBRE84E1DJ20120515>, Reuters.

30. Kurth, A. and A. Siika. *Accuracy of Oral HIV Self-tests in Kenya*. in *3ie Matchmaking Meeting 28 April 2014*. 2014. Nairobi, Kenya.

31. Myers, J.E., et al., *Rapid HIV self-testing: long in coming but opportunities beckon.* AIDS, 2013. **27**(11): p. 1687-95.

32. Kumwenda, M., et al., *Factors Shaping Initial Decision-Making to Self-test Amongst Cohabiting Couples in Urban Blantyre, Malawi.* AIDS Behav, 2014.

33. Balan, I.C., et al., *The Impact of Rapid HIV Home Test Use with Sexual Partners on Subsequent Sexual Behavior Among Men Who have Sex with Men.* AIDS and behavior, 2013.

34. Carballo-Dieguez, A., et al., *Use of a rapid HIV home test prevents HIV exposure in a high risk sample of men who have sex with men.* AIDS and behavior, 2012. **16**(7): p. 1753-60.

35. Drake, A.L., et al., *Incident HIV during Pregnancy and Postpartum and Risk of Mother-to-Child HIV Transmission: A Systematic Review and Meta-Analysis.* PLoS Med, 2014. **11**(2): p. e1001608.

36. Byamugisha, R., et al., *Male partner antenatal attendance and HIV testing in eastern Uganda: a randomized facility-based intervention trial.* Journal of the International AIDS Society, 2011. **14**: p. 43.

37. Becker, S., et al., *Comparing couples' and individual voluntary counseling and testing for HIV at antenatal clinics in Tanzania: a randomized trial.* AIDS and behavior, 2010. **14**(3): p. 558-66.

38. Kiarie, J.N., et al., *Domestic violence and prevention of mother-to-child transmission of HIV-1.* AIDS, 2006. **20**(13): p. 1763-9.

39. Semrau, K., et al., *Women in couples antenatal HIV counseling and testing are not more likely to report adverse social events.* AIDS, 2005. **19**(6): p. 603-9.

40. Kimanga, D.O., et al., *Prevalence and Incidence of HIV Infection, Trends, and Risk Factors Among Persons Aged 15–64 Years in Kenya: Results From a Nationally Representative Study.* JAIDS Journal of Acquired Immune Deficiency Syndromes, 2014. **66**: p. S13-S26 10.1097/QAI.0000000000000124.
